# A Silver Sulfide Cluster with Exterior Diphenylphosphinothioito Ligands Exhibiting a Triskele Motif

**DOI:** 10.1021/acsomega.5c08304

**Published:** 2025-12-02

**Authors:** David M. Rivillo, Robert Burrow, Michele O. Vieira, Henri S. Schrekker, Piet W. N. M. van Leeuwen

**Affiliations:** † Laboratory of Technological Processes and Catalysis, Institute of Chemistry, 28124 Universidade Federal do Rio Grande do Sul, Porto Alegre, Rio Grande do Sul 91501-970, Brazil; ‡ Department of Chemistry, Center for Natural and Exact Sciences, 28118 Universidade Federal de Santa Maria, Santa Maria, Rio Grande do Sul 97105-900, Brazil

## Abstract

A novel silver sulfide cluster Ag_53_S_20_(Ph_2_PS)_24_ has been discovered with cubic shells of Ag and S atoms in the interior but with a unique outer shell consisting of a network of 24 ligands and 20 Ag atoms. The network can be described as a highly distorted octahedron with approximate chiral symmetry O, the eight corners being triskeles of the same symmetry in one molecule (the crystal is racemic: Δ and Λ enantiomers). The outside of the cluster is formed by the protecting hydrophobic 48 phenyl groups. The Ag–Ph_2_PS twisted cube of the Δ enantiomer corresponds closely to the motif of the Brazuca soccer ball. Thus, the cluster presents another rare example of a complex molecule that models a sports ball.

## Graphical abstract

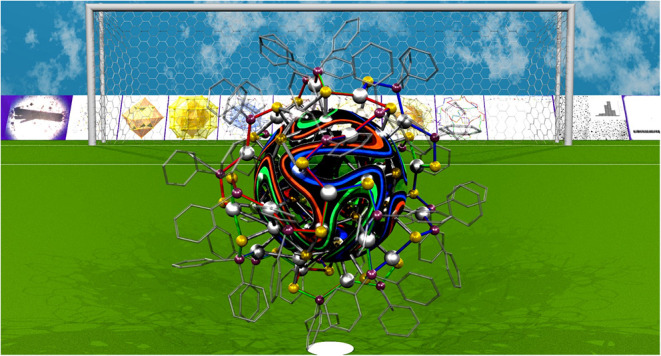

## Introduction

Similarities between molecular structures and macroscopic objects have long been noted in chemistry.^1^ Some are obvious such as ladder polymers,^2^ Möbius strips,^3^ molecular knots,^4^ tweezers,^5^ clips,^6^ baskets,^7^ and zippers^8^ while others require imagination, such as molecular cars,^9^ machines^10^ or wheelbarrows.^11^ Here we show another rare example of a cluster with an outer shell that matches exactly with the motif of the Brazuca soccer ball. A novel silver sulfide cluster Ag_53_S_20_(Ph_2_PS)_24_ has been discovered with cubic shells of Ag and S atoms in the interior but with an outer shell consisting of a network of 24 ligands and 20 Ag atoms. The network can be described as a twisted octahedron with approximate chiral symmetry O, the eight corners being triskeles of the same symmetry in one molecule (the crystal is racemic due to the crystallographic glide plane, containing both the Δ and Λ enantiomers). The Ag–Ph_2_PS twisted cube models the Brazuca soccer ball. Such structural features of this complex molecule may be of interest for future studies on chirality and nanocluster design. Yoshida et al. have shown that it is possible to separate chiral silver clusters into their enantiomers.^12^

A wide variety of nicknames are given to clusters, nanoparticles, and macromolecules, and for cavities in supramolecular chemistry, with a great use of sports balls as molecular models. Buckyball, C_60_ or Buckminsterfullerene, undoubtedly leads the pack of popular molecules, recognized by public as the geodesic domes built by Buckminster Fuller and, more so, as the well-known soccer ball based on Fuller’s design. The Telstar (Figure 1a) ball was used by Adidas for the official play ball of the FIFA soccer world championship in Mexico 1970.^14^ The ball is based on a truncated icosahedron (12 apexes, 20 triangles) consisting of 12 pentagons and 20 hexagons stitched together; having symmetry point group I_h_. The existence of the molecular analogue buckminsterfullerene, C_60_, was predicted in 1970 and it was first synthesized in the 1980s.^13^ Each four years a new ball design was introduced for the championships, but the basic buckyball structure was retained despite the new color designs. The applied designs have no molecular analogues, but for the first newly structured ball, the Teamgeist ball (Germany 2006, Figure 1b), one could imagine a hypothetic molecule W­(N_2_)_6_ with 6 side-bonded dinitrogen molecules having symmetry T_h_.

**Figure 1:**
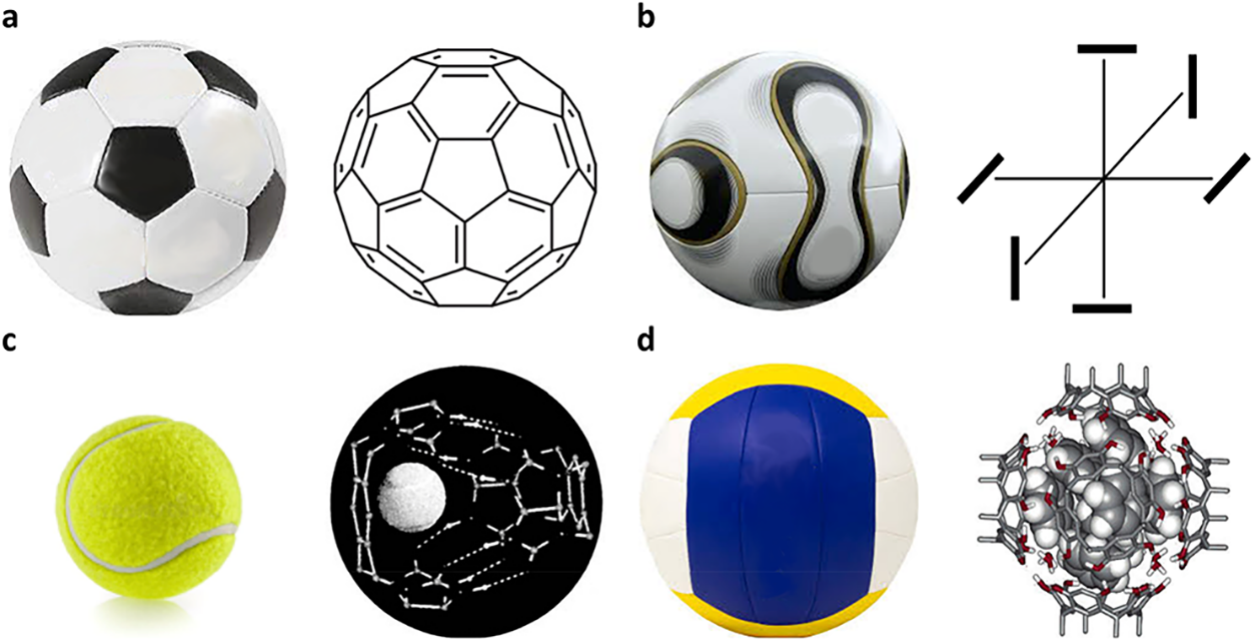
(a) 1970 Telstar, C_60_. (b) 2006 Teamgeist, “W­(N_2_)_6_”. (c) Tennis ball, Rebek tennis ball. (d) Volleyball, Rebek volleyball.

Wyler et al. developed a supramolecular host that modeled a tennis ball or softball (Figure 1c).^14^ A supramolecular arrangement of six hydrogen bonded resorcinarenes with an approximate O_h_ symmetry was named volleyball, but the usual structure of a volleyball has a different structure and symmetry, T_h_ (Figure 1d).^15^ Hexameric cages of six calix[4]­arene tetracarboxylates and eight uranyl cation units UO_2_^2+^ gave assemblies of perfect O_h_ symmetry.^16^

During our work on metal nanoparticles of Au, Ag, and Ir ligated by phosphinito and phosphinothioito ligands we accidentally hit upon an interesting structure in the context of modeling sports balls by molecular structures.

## Results and Discussion

Metal nanoparticles of Au, Ag, and Ir ligated by phosphinito and phosphinothioito ligands have properties resembling those of the ubiquitous alkyl/aryl sulfides in heavy metal cluster chemistry, but interestingly they have two donor atoms, P–O or P–S giving rise to different bonding, and they can assist in heterolytic cleavage of hydrogen in catalytic reactions.^17–20^ Thus, using the successful ligand exchange recipe of Bakr we treated Na_4_[Ag_44_(MNBA)_30_] nanoclusters ligated by water-soluble 5-mercapto-2-nitrobenzoic acid (MNBA) ligands with an organic phase of dichloromethane containing diphenyl-λ^5^-phosphanothione.^21^ Visually the ligand exchange reaction took place. UV–Vis spectroscopy shows that the parent aqueous Na_4_[Ag_44_(MNBA)_30_]·30Na (bands at 388, 483, 558, 651 nm) was consumed during the biphasic ligand-exchange: after extraction the organic phase displays new, broader absorptions at 460, 508, and 687 nm (Supporting Information, Figure S1). The disappearance of the Ag_44_ fingerprint bands together with the appearance of these red-shifted features indicates formation of a chemically transformed Ag–S cluster with a different electronic structure rather than simple phase transfer. This interpretation is consistent with the concurrent ^31^P NMR detection of Ph_2_PP­(S)­Ph_2_ (Supporting Information, Figure S2), evidencing oxidation of Ph_2_P­(S)H and participation of the phosphinothione in cluster formation. This cluster was further characterized through transmission electron microscopy (Supporting Information, Figure S3) and thermogravimetric analysis (Supporting Information, Figure S4). TEM revealed well-dispersed, roughly spherical nanoparticles with a mean diameter of 2.9 ± 0.8 nm, consistent with nanocluster formation. TGA/DTG analysis showed a multistep weight loss: a minor initial loss (∼3.2% up to 167 °C) corresponding to solvent and surface species, followed by a major decomposition event between 200–400 °C (DTG peak at 360 °C) assigned to ligand degradation. The final residue of ∼41.3% at 900 °C is consistent with the silver sulfide/metallic silver core. Its Fourier-transform infrared spectrum (Supporting Information, Figure S5) shows an adsorption at 543 cm^–1^, typical of bridging R_2_PS (540–585 for Rh and Ir­(I)).^22^ In solution the formation of Ph_2_PP­(S)­Ph_2_ was observed by ^31^P nuclear magnetic resonance spectroscopy (Supporting Information, Figure S2) indicating that Ag(0) had been oxidized.^23^ In matrix-assisted laser desorption/ionization-time-of-flight mass spectrometry no MWs for parent cluster molecules as obtained after recrystallization were observed (Supporting Information, Figure S6), which may be due to the high charge. After workup dark violet crystals (Supporting Information, Figure S7) were obtained that analyzed for Ag_53_S_20_(Ph_2_PS)_24_ (Na/K)_11_(THF)_*x*_ **1** and crystallized in the monoclinic space group “*P*2/*c*” with 4 molecules in the unit cell. An overall picture of one molecule is shown in Figure 2. The spherical nature of **1** gives rise to a hexagonal close packing (HCP) type arrangement in the crystal, with the total volume of the cell occupied by 87.1% of **1** and the remainder, 12.9% occupied by solvent and cations (Supporting Information, Figure S8).

**Figure 2:**
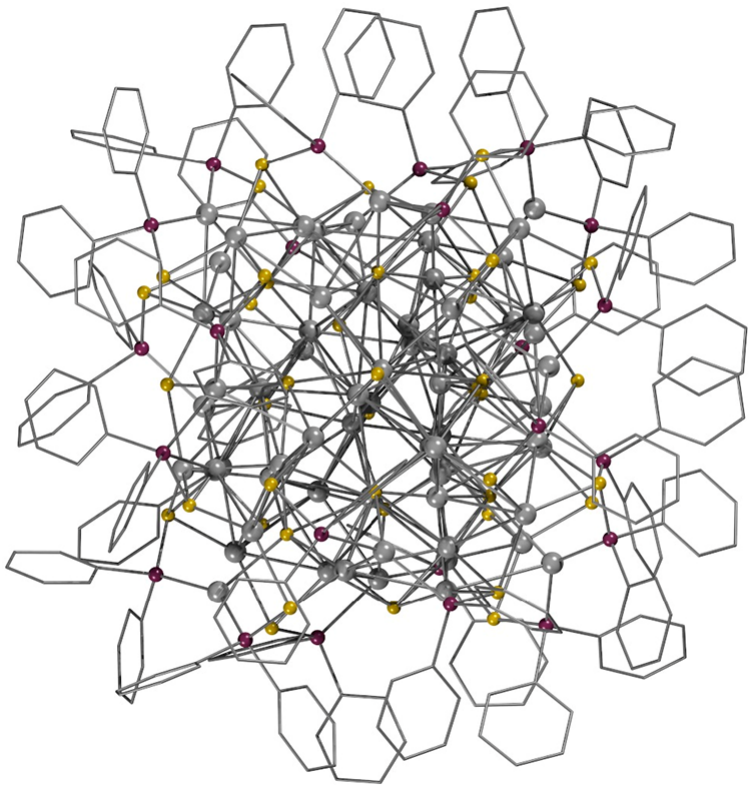
Structure of 1: gray = Ag; yellow = S; purple = P; gray hexagons = C_6_H_5_ (Supporting Information, Video S1).

The Na/K cations and THF molecules could not be localized in the electron density map, which is not uncommon for cations, anions, and solvents in clusters of this size, and the diffuse electron density was removed for better refinement.^16–24^ Nanoparticles with a core of Ag_2_S (Se, and Te) molecules have been widely synthesized as their spectroscopic and electronic properties are subject of investigation.^25^ Their sizes range from 7 to 490 Ag atoms and the outside is protected by thiolates, selenolates, tellurates, phosphines, etc. This is the first nanocluster in which a phosphanothione (R_2_P­(S)­H) both forms the stabilizing ligand, diphenylphosphinothioito, and provides sulfide S^2–^ anions for Ag_2_S during the synthesis, in the same way as was observed for thiolate ligands.^26^ We propose the following stoichiometry for the formation of the Ag_53_ cluster, without mechanistic implications: 1.2 Na_4_[Ag_44_(MNBA)_30_]·30Na + excess Ph_2_P­(S)H + excess KOH → [Ag_40_S_20_]­[Ag]­[Ag­(Ph_2_PS)_2_]_12_·11K/Na + 20 Ph_2_PP­(S)­Ph_2_ + 11 H_2_ + 36 MNBA·2Na/K (Supporting Information, Text S1).

The outside layers of **1** consist of 24 diphenylphosphinothioito ligands, 12 sulfide anions, and 20 Ag atoms arranged in a highly regular, symmetric fashion that can be described as a series of embedded regular polyhedra, vide infra. The outside 20 Ag atoms form a cuboctahedron (Ag02, Ag04, Ag06, Ag08, Ag09, Ag10, Ag11, Ag12, Ag14, Ag16, Ag18 & Ag20) interpenetrated by a cube (Ag01, Ag03, Ag05, Ag07, Ag13, Ag15, Ag17 & Ag19) such that the Ag atoms of the cube cap the triangular faces of the cuboctahedron, shown in Figure 3. This creates a partially stellated polyhedron, which has 20 vertices, six square faces and 24 triangular faces (approximate symmetry Oh). The eight Ag atoms of the cube are bordered by three triangular faces while 16 Ag atoms form part of two square faces and four triangular faces. This arrangement generates 24 edges with the Ag···Ag distances ranging in length from 3.964(3) to 4.431(3) Å.

**Figure 3:**
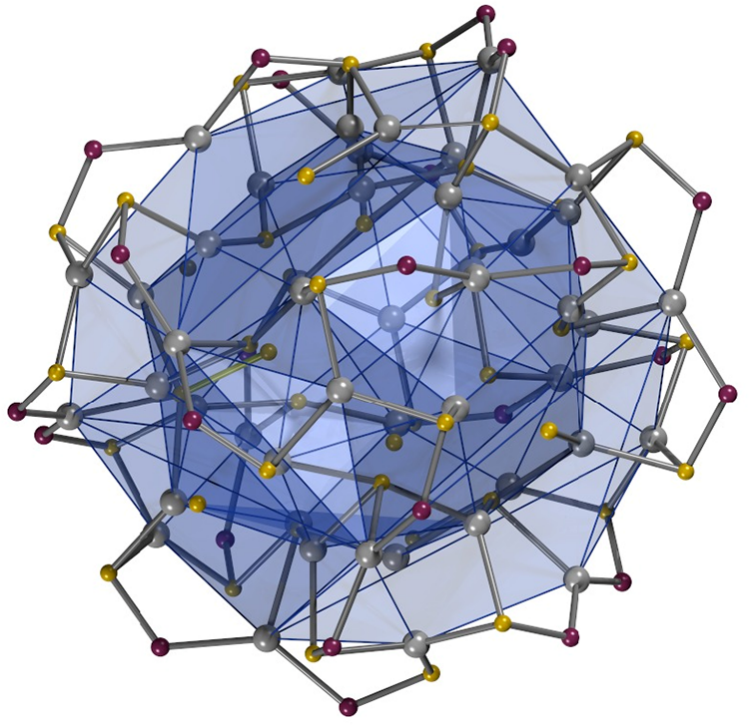
Outside and inside polyhedra formed by the Ag atoms in 1 (Supporting Information, Figure S9; Supporting Information, Video S2).

Outside the first shell of Ag atoms are the 24 diphenylphosphinothioito ligands which bind in a bridging fashion with their S atoms (S1–S24) to the eight Ag atoms of the cube, with three S atoms per Ag atom, and their P atoms (P1–P24) to the 12 Ag atoms of the cuboctahedron, with two P atoms per Ag atom. The three S atoms per Ag atom respect the approximate 3-fold symmetry of the vertex of the Ag polyhedron forming approximate isosceles triangles, with the Ag atom approximately coplanar with the triangle of S atoms, to form a rhombicuboctahedron (24 vertices, 18 square faces and eight triangular faces; Figure 4a). The P atoms of two diphenylphosphinothioito ligands bind to each of the 16 Ag atoms (the remainder of Ag01–Ag24), with P–Ag–P angles varying from 128° to 140°, to form a snub cuboctahedron (24 vertices, six square faces and 32 triangular faces). The arrangement of the Ag–P–S–Ag–S–P–Ag bonds form an 8-fold triskele pattern very much like the Adidas Brazuca ball used in the soccer world cup of 2014, held in Brazil, vide infra. The forty-eight phenyl groups of the diphenylphosphinothioito ligands make up the outside hydrophobic shell of the cluster.

**Figure 4:**
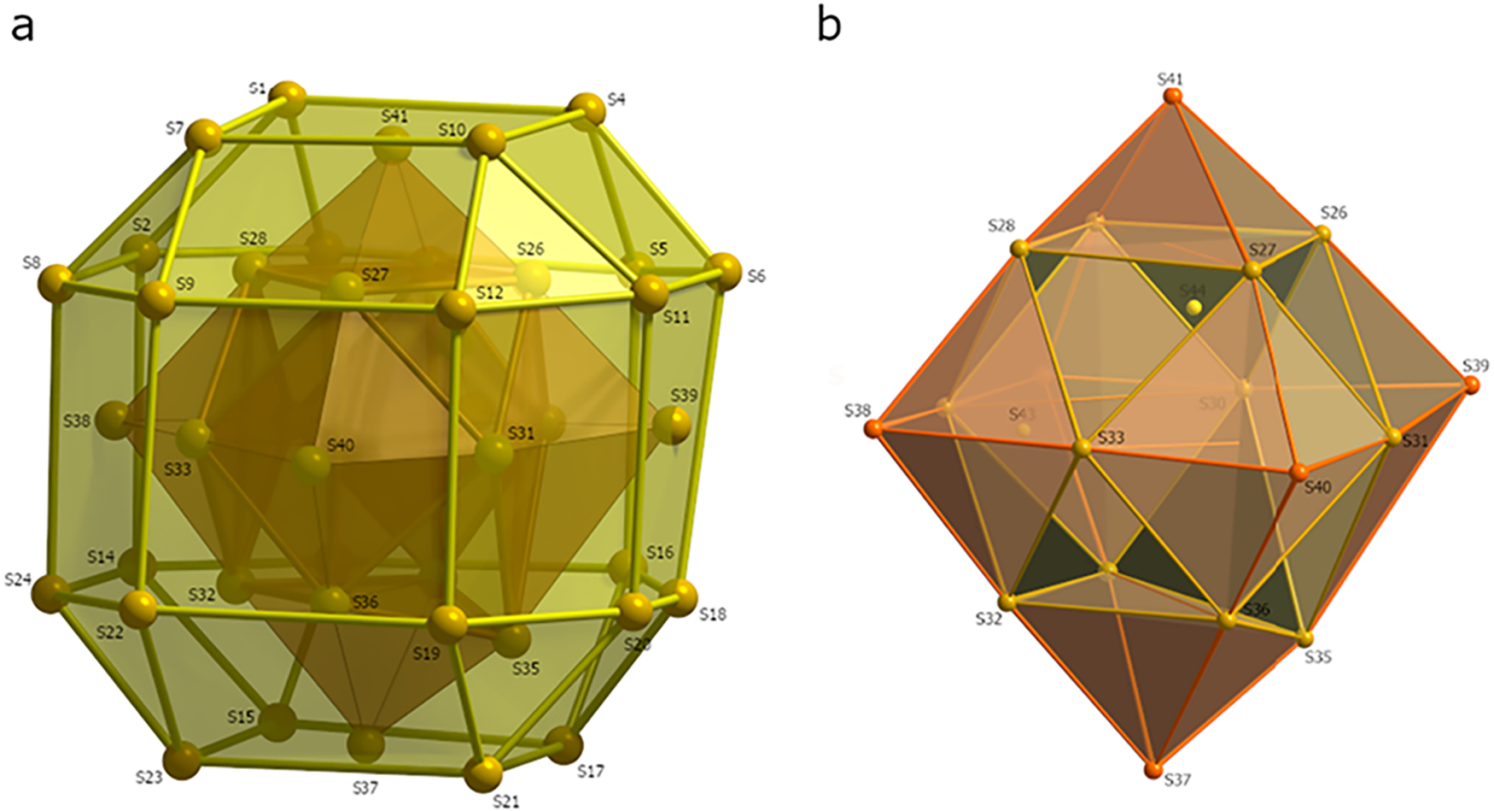
Rhombicuboctahedron formed by the 24 S (S1–24) atoms of the Ph_2_PS ligands (Supporting Information, Figure S10; Supporting Information, Video S3) (a) and the arrangement of the 20 sulfide ions in 1 (Supporting Information, Figure S11; Supporting Information, Video S4) (b).

Inside the first shell of Ag atoms are additional 18 S^2–^ ions (S25–S42). Twelve of these (S25–S36) form a cuboctahedron which is interpenetrated by an octahedron formed by the remaining six (S37–S42) S atoms while the square faces of the cuboctahedron are capped by the vertices of the octahedron to form a partially stellated polyhedron with 18 vertices and 32 triangular faces (Figure 4b). Inside the cuboctahedron of S atoms, a distorted rhombicuboctahedron can be found, made up of 24 Ag atoms, most of which (Ag21–Ag39) are not disordered, and some (Ag41, Ag42, Ag44, Ag59 & Ag60) have refined to partial occupancy. Inside the Ag rhombicuboctahedron are one fully occupied Ag atom (Ag40), twenty-one partially occupied Ag atoms (Ag43, Ag45–Ag58), and the remaining two sulfide anions (S43 & S44). The two sulfide atoms refined well to full occupancy, whereas a trial with partial occupancy Ag atoms led to poor refinement. Each partially occupied Ag atom with electron density less than about 16 *e* ^–^ Å^–3^ (about 0.37 occupancy) was tested with refinement as a partially occupied S atom but did not refine well. It is noteworthy that although no restraints nor constraints were applied to the occupancy of the partially occupied Ag atoms, the refinement converged to a total of 13.00 Ag atoms for the twenty-six partially occupied Ag atoms. The partially occupied Ag atoms were refined in two parts: Ag41–Ag56, with occupancies of 0.932–0.545, which are reasonably far from their neighbors, and Ag57–Ag66, with occupancies of 0.473–0.136, which are located close to the Ag atoms in the first part. Together with the 40 fully occupied Ag atoms, there are fifty-three Ag atoms in total, as shown in the formula of 1.

The aspect most intriguing is that the edges thus consist of Ag–S–P–Ag–P–S–Ag sequences, apexes included. Importantly, the edges are not linear but S-shaped, which one can envisage brought about as a twist around the approximate 3-fold symmetry axis at each apical Ag atom. This rotation is done in the same direction for all 8 apexes, which results in a chiral molecule of approximate symmetry O (crystallographically the symmetry is formally lower; also, the crystals are racemic as both enantiomers are present, Δ and Λ). Chirality in nanoclusters^27^ has been reported before for Au with chiral^28^ and nonchiral ligands,^29,30^ and Ag.^4,12,31^ E.g., the latter example of atomically precise and intrinsically chiral nanocluster [Ag_78_(DPPP)_6_(SR)_42_], where DPPP is the achiral 1,3-bis­(diphenylphosphino)­propane and SR = SC_6_H_4_CF_3_, crystallized as racemates in a centric space group. The cubic Ag–P–S network of 1 is depicted in Figure 5a,b and as Crassous did, we named this the right-handed enantiomer, Δ.^32^ Nakashima uses clockwise (C) for this hand.^12,33^ The Δ enantiomer also exists within the unit cell as it is generated by the crystallographic *c* glide. This motif corresponds closely to the panel design of the Brazuca soccer ball (Figure 5c).^34^ The topology of the outer shell of the cluster can be conveniently illustrated by comparison with the panel design of the Brazuca soccer ball. In the nanocluster, the network of 24 SPS and 8 + 12 Ag atoms are the borders of the six faces (the panes of Brazuca) that constitute the ball; the six panes are the distorted faces of the cubes, obtained by twisting all 8 vertices in the same direction. When these faces of a cube project onto the sphere of the ball, the eight 3-fold vertices give the triskele motif.

**Figure 5:**
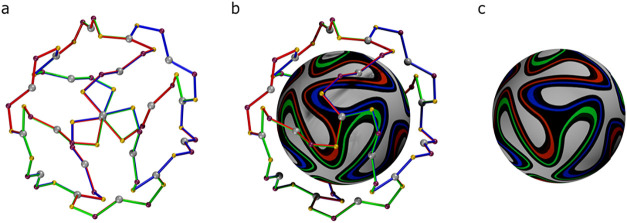
Motif formed by the P and S ligand atoms and the outer 20 Ag atoms in cluster 1 (a, b) and the FIFA championship soccer ball, Brazuca, Adidas, Brazil 2014 (b, c) (Supporting Information, Figures S12 and S13; Supporting Information, Video S5). The bicoloured bonds correspond to the panel colors of the ball.

In the Brazuca ball, the panel arrangement is structural, not merely decorative, providing a useful macroscopic analogy for the twisted cube geometry observed in the cluster. The resulting grooves are important for the aerodynamics of the ball; the larger the total volume of the grooves, the longer turbulent flow is retained during the flight and the more stable the flight is.^26,35,36^ Spinning of the ball also adds to the stability of the flight, albeit curved,^37^ and spinning should interact with the chiral structure. In the numerous studies on aerodynamics of sports balls this seems to have received little attention.^38^ A search for images of the Brazuca ball shows that the commercial versions consistently exhibit the Δ (right-handed) isomer.

## Methods

### Materials

Reagents were purchased from Sigma-Aldrich (Merck) and used without further purification. CH_2_Cl_2_ and ethanol were HPLC grade and Milli-Q water was used throughout this study.

### Characterizations

The size of the nanoparticles was studied by transmission electron microscopy with a JEOL JEM 1011 CX-T electron microscope working at 100 kV (LPCNO-INSA, Toulouse, France). The grid was prepared by dropping a THF-diluted solution of the nanoparticles onto a nickel grid. Nuclear magnetic resonance spectra were recorded in a 400 MHz Varian spectrometer at ambient probe temperature and referenced to internal tetramethylsilane. The Fourier-transform infrared analysis was performed in a Bruker α-P spectrometer. Thermogravimetric analysis was performed in a Q50 TA equipment operating under a nitrogen gas atmosphere. The sample (10 mg) was heated under N_2_ (25 L/min) from 24 to 900 °C with a heating rate of 10 °C/min. Ultraviolet–visible spectra were recorded in NaOH 1 M or CH_2_Cl_2_ using freshly prepared samples in a 2440 Shimadzu spectrometer. Matrix-assisted laser desorption/ionization-time-of-flight mass spectrometry analysis was performed by using a Bruker Autoflex Speed instrument. The nanoparticle solution in CH_2_Cl_2_ (3 mg/mL) was mixed with the matrix trans-2-[3-(4-tertbutylphenyl)-2-methyl-2-propenylidene]­malononitrile (10 mg/mL in DCM) in the volume ratio of 1:1000. Then, the prepared solution (5 μL) was then spotted on a matrix-assisted laser desorption/ionization target plate and dried completely under ambient conditions before analyzing.

### Synthesis of Ph_2_P­(S)­H

Diphenyl-λ^5^-phosphanothione was synthesized using a modified version of the procedure described by Semenzin *et al*.^39^ To a solution of diphenylphosphine (7.7 g, 41.4 mmol) in 60 mL of dried toluene was added S_8_ (1.38 g, 43.4 mmol) under argon. The solution was heated at 70 °C for 3 h, and the solvent was removed under vacuum. 5 mL of ethanol was added to the residue and stirred until a white product was formed. The ethanol was removed under vacuum and the white residue was recrystallized from a mixture of methanol/water (4:2) to yield 3.3 g (38%) of white crystals. ^31^P NMR (162 MHz, CDCl_3_) δ 20.9 (d, ^1^ *J*_HP_ = 467 Hz); ^1^H NMR (400 MHz, CDCl_3_) δ 8.0 (d, ^1^ *J*_HP_ = 467 Hz, 1H), 7.8–7.4 (m, 10H).

### Synthesis of Na_4_[Ag_44_(MNBA)_30_]·30Na

Na_4_[Ag_44_(MNBA)_30_]·30Na was synthesized according to the procedure described by AbdulHalim *et al*.^21^

### Ligand Exchange

Brazuca nanocluster 1 was prepared by a modified version of the ligand exchange procedure described by AbdulHalim *et al*.^21^ 2 mL of H_2_O containing 2 mg (8.7 μmol, 10 equiv) of benzyltriethylammonium chloride was added to a solution of 10 mg (0.87 μmol, 1 equiv) of Na_4_[Ag_44_(MNBA)_30_]·30Na cluster in 3.8 mL of KOH 1M. Attempts to use NaOH instead of KOH did not yield crystalline material, indicating that KOH is crucial under these conditions for obtaining the Ag_53_S_20_(Ph_2_PS)_24_ cluster. Then 7.4 mg of Ph_2_P­(S)H (0.034 mmol, 39 equiv) dissolved in 9 mL of CH_2_Cl_2_ was added to the above mixture. The two phases were vigorously mixed for 2.5 min, and the change of the organic layer to black and the aqueous layer to clear yellow confirmed the exchange of ligands. The organic layer was separated and passed through a column with CaCl_2_–MgSO_4_–Celite. The solvent was evaporated, and the residue was washed with ethanol three times to remove the excess ligands. Product was dried under high vacuum to get 3.5 mg of dark brown powder (50–60% yield). Despite extensive crystallization attempts using vapor diffusion, liquid–liquid diffusion, and controlled evaporation with various solvent systems (acetone, CH_2_Cl_2_, toluene, THF with pentane or hexane), only a large needle-shaped crystal obtained from CH_2_Cl_2_/pentane was suitable for single-crystal X-ray diffraction. This dark violet crystal was obtained by diffusion of pentane into a CH_2_Cl_2_ solution of nanoclusters at room temperature (1–2 days), which was subjected to X-ray diffraction.

### Reproducibility of the Cluster Formation

The synthesis of the Ag_53_S_20_(Ph_2_PS)_24_ cluster was reproducibly achieved under the same reaction conditions described above. Each batch consistently yielded a dark violet product exhibiting identical FTIR transmittance features to the original sample. However, only one crystal displayed sufficient quality for single-crystal X-ray diffraction; subsequent crystallizations typically produced amorphous or microcrystalline solids that did not diffract. These observations indicate that the cluster forms reproducibly, while the growth of diffraction-quality crystals remains challenging and stochastic, as often encountered for large silver sulfide assemblies.

### X-ray Crystallography

The reported structure was obtained from a single crystal that initially diffracted well; however, upon subsequent measurement attempts the crystal no longer diffracted, suggesting structural changes. Repeated crystallization did not reproduce this crystal. A Bruker D8 Venture Photon 100 CCD diffractometer system equipped with an Incoatec Montel two-dimensional X-ray optics monochromator and a Mo Kα microfocus X-ray tube (λ = 0.71073 Å) and an Oxford Cryosystems CryoStream 800 was used for the 100 K data collection. Data were integrated using SAINT; absorption correction performed with SABADS; the solution made with Bruker XT by intrinsic phasing; and the refinement with full-matrix least-squares in Bruker XL, all within the APEX environment, which produced the tables and final crystallographic information file. PLATON was used for the SQUEEZE treatment which removed the nebulous electron density, corresponding the greatly disordered counterions and solvate molecules, in the cell. The void space in the unit cell is shown in Figure S14 (Supporting Information) and the Ag atom disorder is shown in Figure S15 (Supporting Information). Diamond (Crystal Impact GbR, v.3.2k) was used to produce the initial structure diagrams which were exported to Povray input files. Povray v3.8 was used to produce the final diagrams from the heavily edited files. The crystallographic data for 1 can be obtained under the deposition number 2225255, free of charge, from the Cambridge Crystallographic Data Centre (http://www.ccdc.cam.ac.uk/data_request/cif).

### Crystallographic Data for (1), C_288_H_240_P_24_S_44_Ag_53_

Dark violet plates, crystal size 0.05 × 0.19 × 0.30 mm, *M* = 11,571.82 g mol^–1^, *F*_000_ = 22092, monoclinic space group, *P*2/*c*, *a* = 40.070(7) Å, *b* = 23.150(4) Å, *c* = 38.910(7) Å, *V* = 36009(11) Å^3^, *Z* = 4, *D*_calcd_ = 2.134 Mg m^–3^, μ­(Mo Kα) = 3.210 mm^–1^, 1 011 830 collected reflections, 2.12 ≤ θ ≤ 28.37°; −53 ≤ *h* ≤ 53, −30 ≤ *k* ≤ 30, −51 ≤ *l* ≤ 51, 89 620 independent reflections, *R*_int_ = 0.166, 99.4% coverage, *R*_1_ = 0.198 5 and w*R*_2_ = 0.444 2 for *I* ≥ 2σ­(I), *R* = 0.239 2 and w*R*_2_ = 0.467 4 for all reflections, *S* = 1.088, and the largest difference hole and peak are −4.60 and 8.75 *e* ^–^ Å^–3^.

## Conclusions

The scope of intricate molecules that model sports balls has been expanded with the novel silver sulfide cluster Ag_53_S_20_(Ph_2_PS)_24_. Remarkably, the outer shell exhibits approximate chiral symmetry O, forming an Ag–Ph_2_PS twisted cube that mirrors the motif of the Brazuca soccer ball. Beyond this visual analogy, the cluster provides several insights of broader significance. It represents a structurally novel silver sulfide assembly stabilized by diphenylphosphinothioito ligands and displays intrinsic chirality, crystallizing as a racemate of Δ and Λ enantiomers. The synthesis further highlights the dual role of diphenylphosphinothione, serving both as a coordinating ligand and as a source of sulfide anions. Taken together, these features extend the importance of this work beyond the molecular–soccer ball analogy, offering new perspectives for nanocluster design and potentially inspiring future studies in catalysis and materials science. The structural analogy with the Brazuca ball provides a striking visual comparison but is presented here primarily to aid understanding of the cluster’s geometry.

## Supplementary Material

















## Notes

Supporting Figures S1 to S15 and Text S1 (PDF)
Supporting Videos S1 to S5 (MP4)­(MP4)­(MP4)­(MP4)­(MP4)
RB_UFRGS_HS_GM79_100K_Mo_Deposit (CIF)
Check CIF (PDF)
