# Supplementary material for: A Silver Sulfide Cluster with Exterior Diphenylphosphinothioito Ligands Exhibiting a Triskele Motif
Source: ACS Omega. 2025 Dec 2;10(49):60572–8. doi: 10.1021/acsomega.5c08304 (PMC12713494; doi:10.1021/acsomega.5c08304)
Supplement: Supplementary file 1 [file ao5c08304_si_001.pdf]

SUPPORTING INFORMATION

A Silver Sulfide Cluster with Exterior Diphenylphosphinothioito Ligands Exhibiting a Triskele Motif

David M. Rivillo,[†] Robert Burrow,[‡] Michele O. Vieira,[†] Henri S. Schrekker[*,†] & Piet W. N. M. van Leeuwen[*,†]

[1] Laboratory of Technological Processes and Catalysis, Institute of Chemistry, Universidade Federal do Rio Grande do Sul, Porto Alegre, RS, 91501-970, Brazil.

[2] Department of Chemistry, Center for Natural and Exact Sciences, Universidade Federal de Santa Maria, Santa Maria, RS, 97105-900, Brazil.

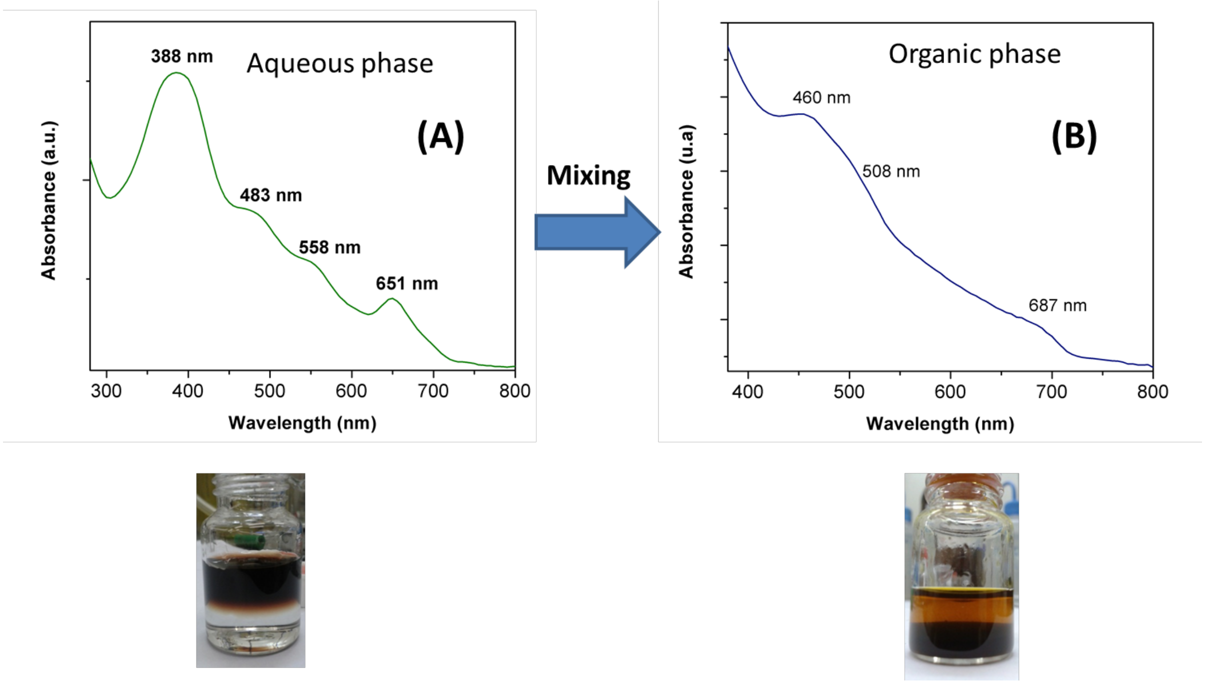

Figure S1. (A) Ultraviolet–visible absorbance spectrum of the aqueous phase of $Na_4[Ag_{44}(MNBA)_{30}]\cdot 30Na$ before ligand exchange, with characteristic absorption bands at 388, 483, 558, and 651 nm. Inset: photograph of the biphasic system prior to mixing. (B) Ultraviolet–visible absorbance spectrum of the organic phase after ligand exchange, with bands at 460, 508, and 687 nm. Inset: photograph of the biphasic system after mixing.

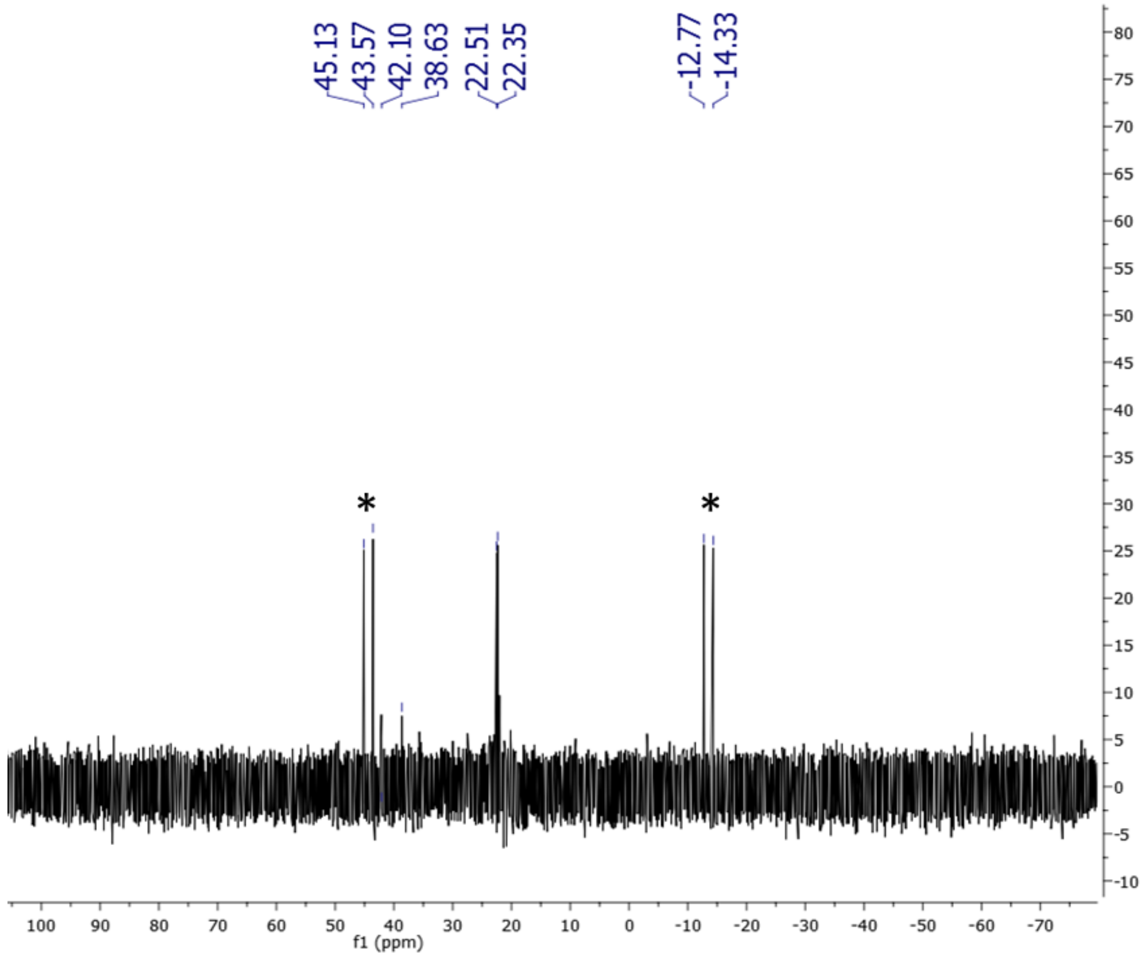

Figure S2. ^{31}P nuclear magnetic resonance (NMR) spectrum (162 MHz, CD_2Cl_2) of the organic phase after ligand exchange: * = $Ph_2P(S)PPh_2$, δ 44.38 (d, J = 252 Hz) and −13.52 (d, J = 252 Hz). Spectrum plotted as intensity (a.u.) versus chemical shift δ (ppm).

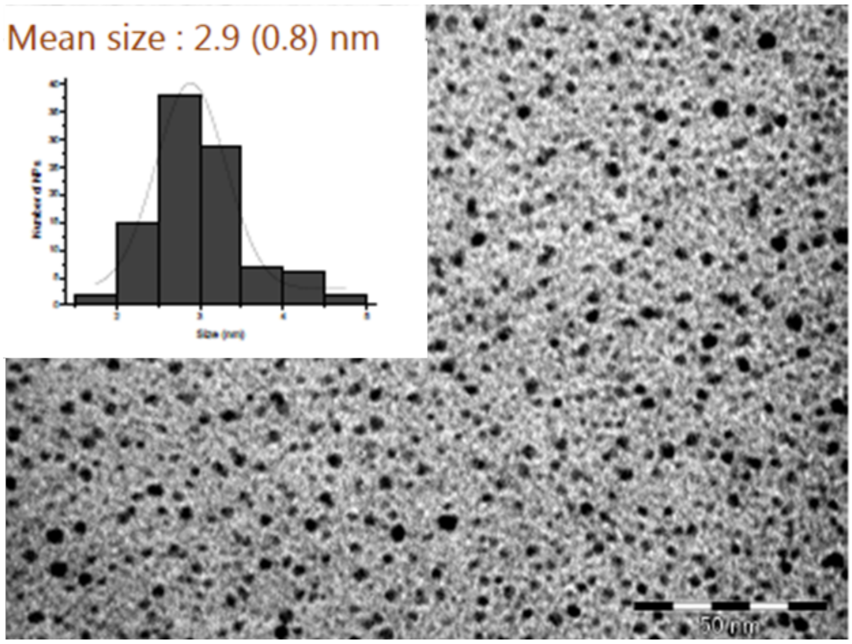

Figure S3. Transmission electron micrograph of the ligand-exchanged product (scale bar = 50 nm). Inset: nanoparticle size distribution histogram with a mean diameter of 2.9 ± 0.8 nm.

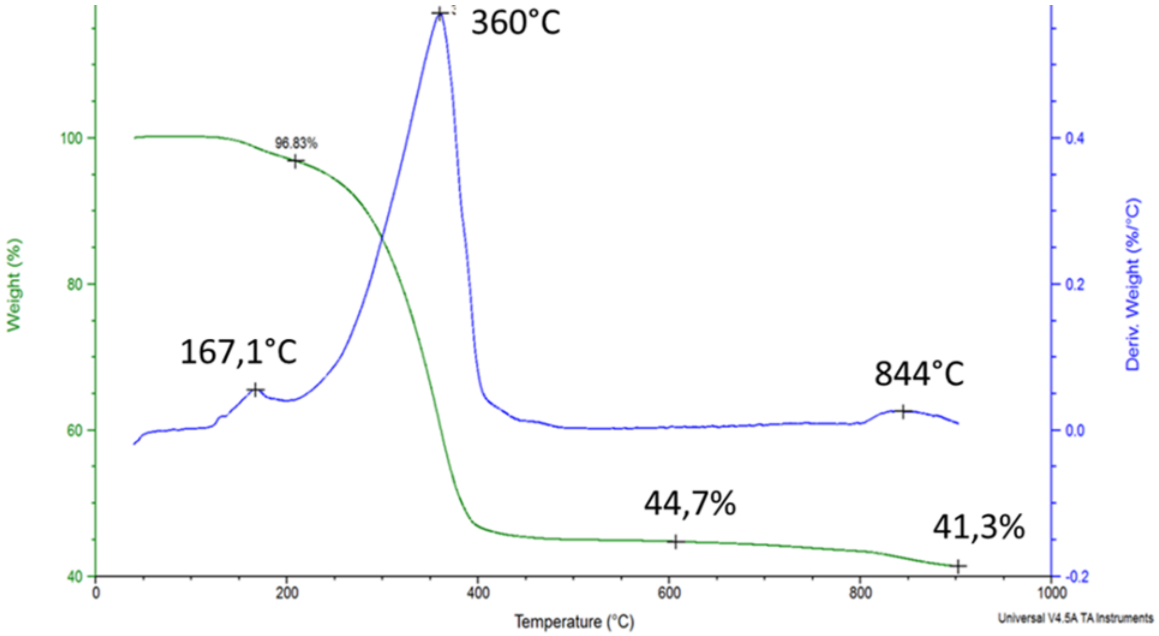

Figure S4. Thermogravimetric (green) and derivative thermogravimetric (blue) curves of the ligand-exchanged product, showing multistep mass loss with a final residue of ~41.3%.

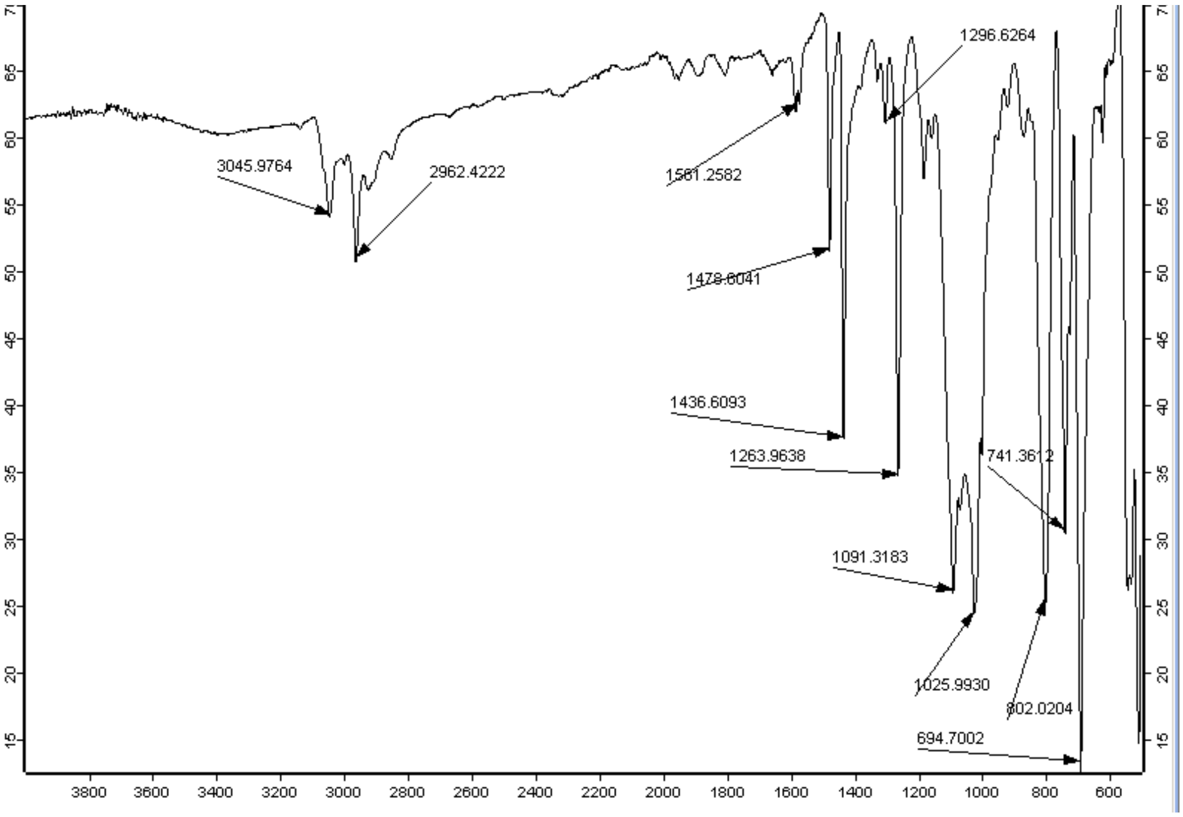

Figure S5. Fourier-transform infrared (FTIR) spectrum of the ligand-exchanged product, showing transmittance (%) versus wavenumber (cm^{-1}).

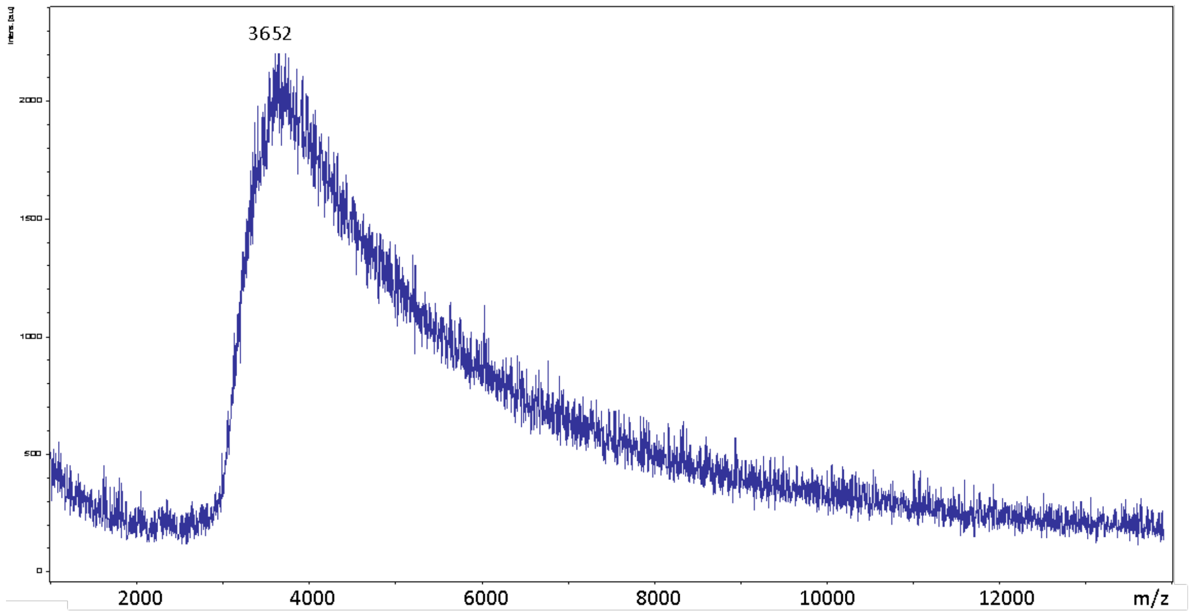

Figure S6. Matrix-assisted laser desorption/ionization-time-of-flight (MALDI-TOF) mass spectrum of ligand-exchanged product in linear positive ionization mode. The most intense signal corresponds to m/z ~3652 Da. Spectrum plotted as intensity (a.u.) versus m/z.

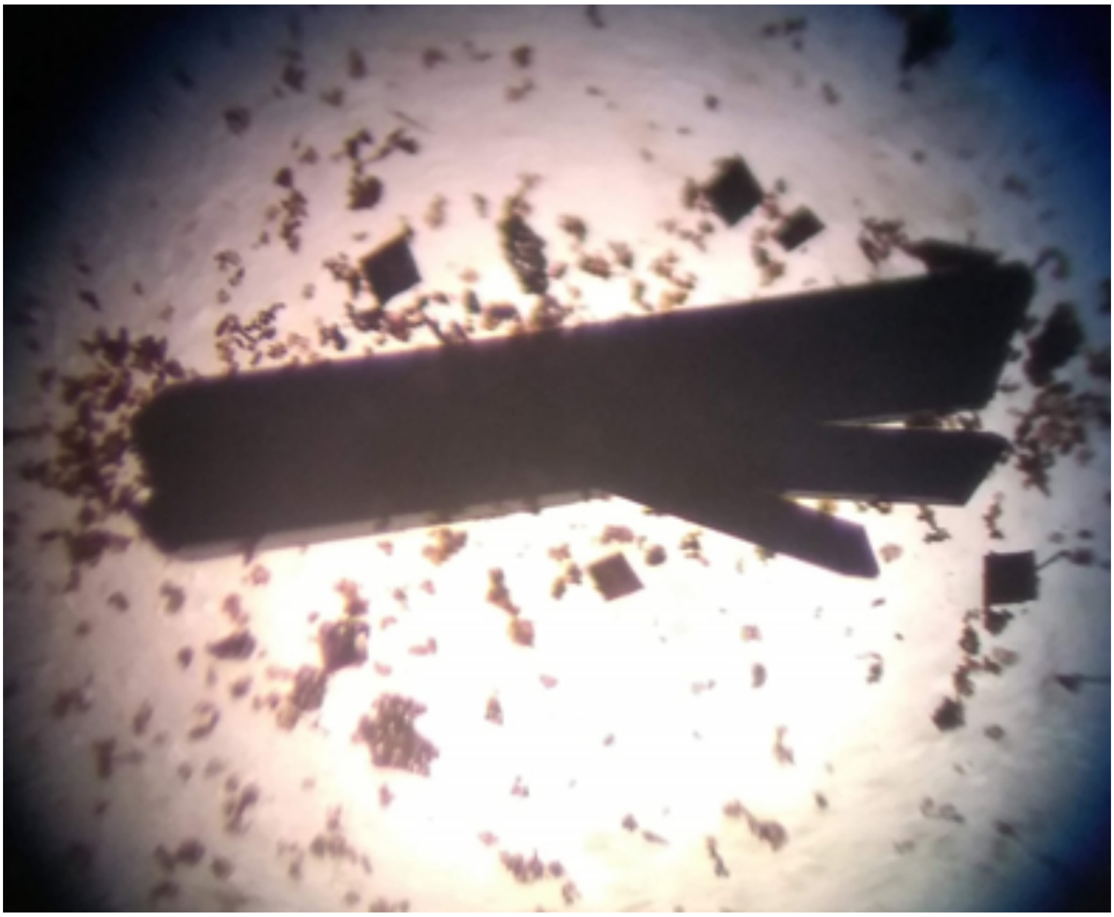

Figure S7. Optical micrograph of dark violet crystals. Crystallization attempts were extensively carried out using vapor diffusion, liquid–liquid diffusion, and controlled evaporation with various solvent systems (acetone, CH_2Cl_2, toluene, THF with pentane or hexane), but only the large needle-shaped crystal obtained from CH_2Cl_2/pentane provided suitable diffraction data that corresponded to $Ag_{53}S_{20}(Ph_2PS)_{24}$. Smaller block-like crystals and other conditions consistently failed to diffract properly.

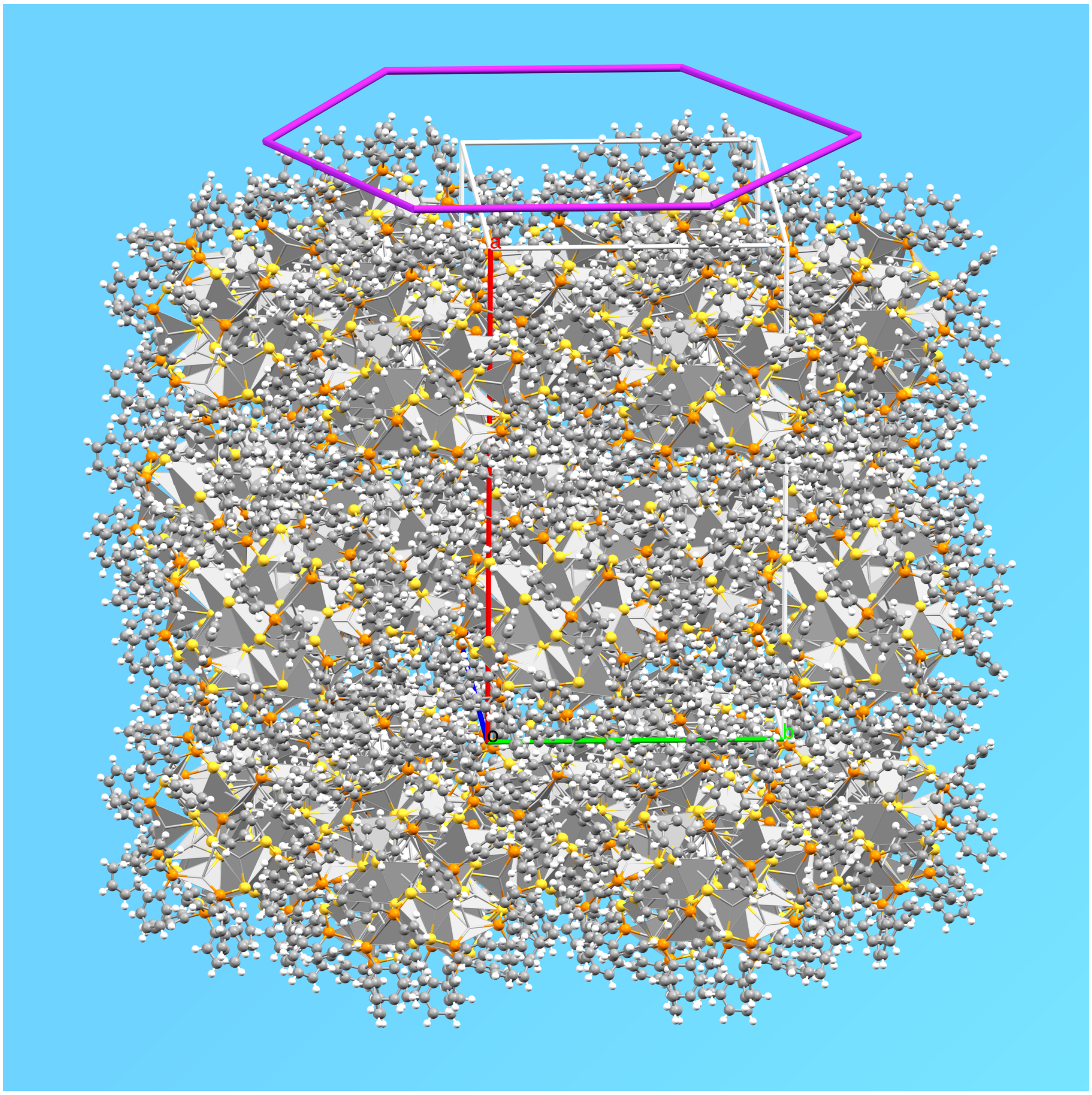

Figure S8. Packing diagram of **1**, showing the hexagonal close packing, indicated by the purple hexagon as a visual aid.

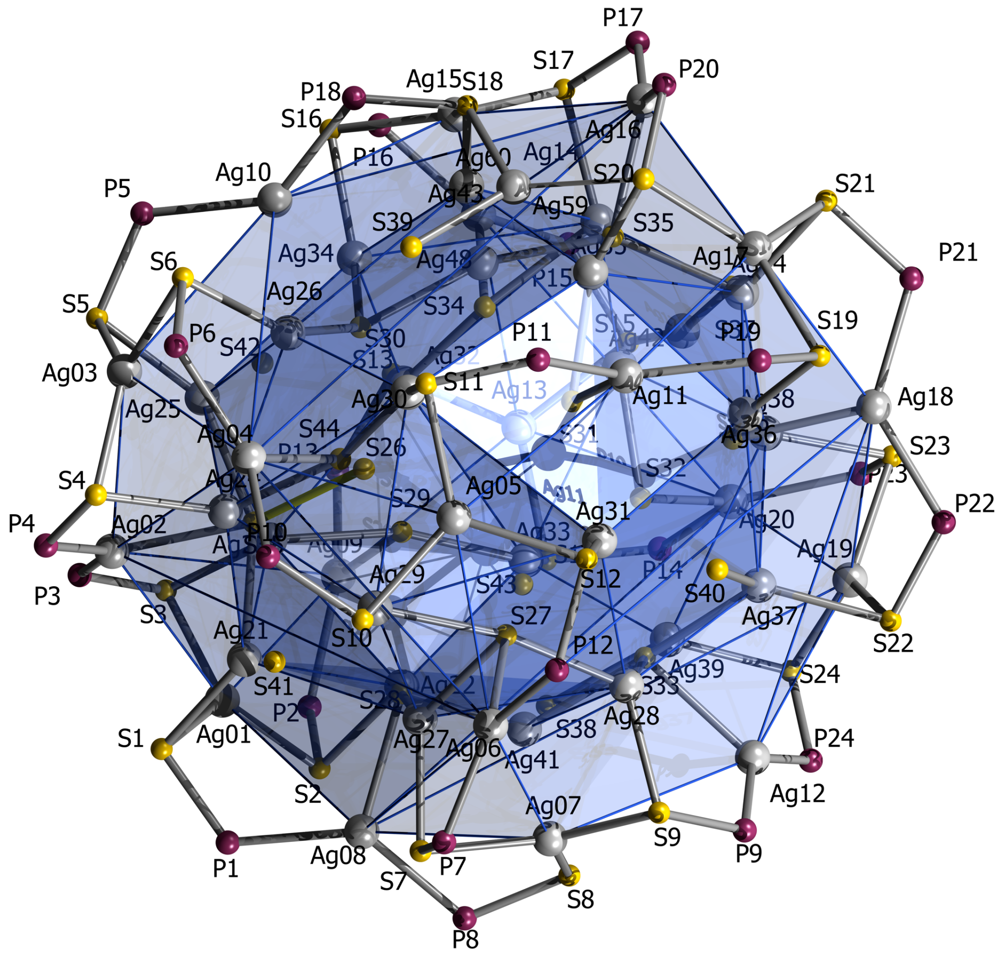

Figure S9. The outside and inside polyhedra formed by the Ag atoms in **1**.

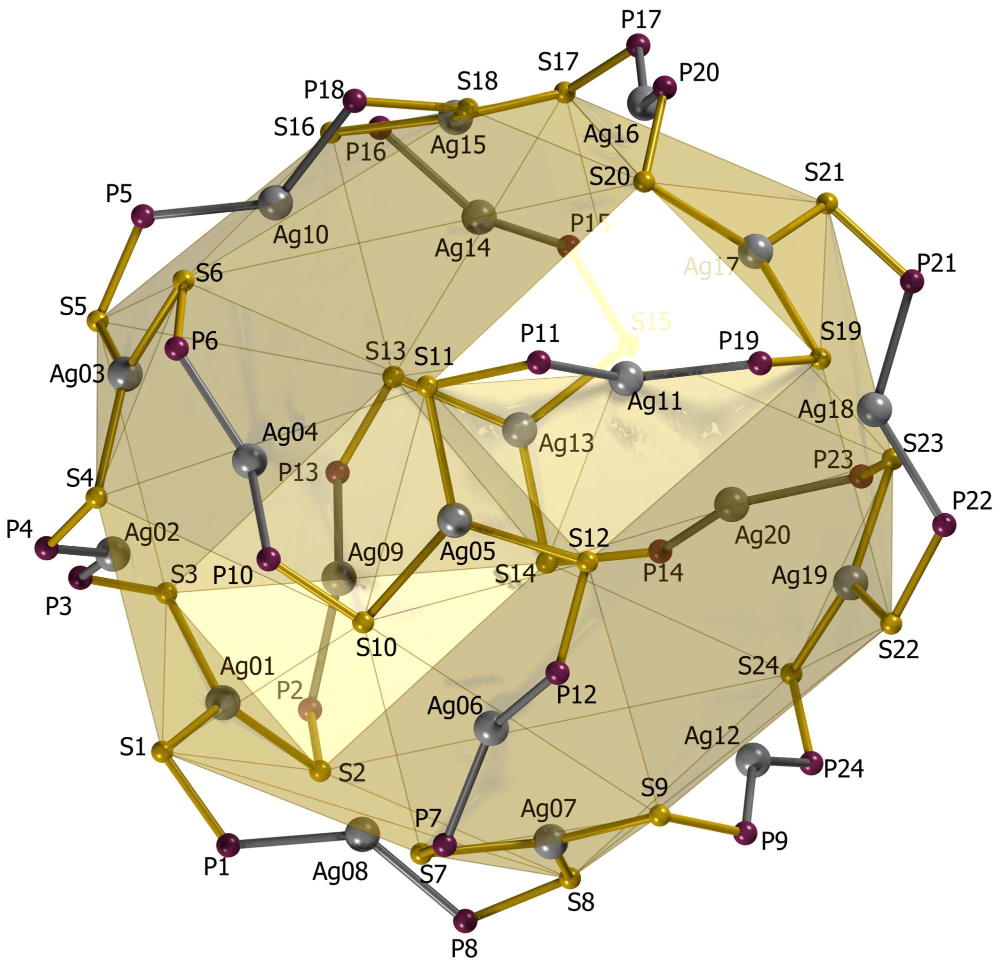

Figure S10. The rhombicuboctahedron formed by the 24 S (S1–24) atoms of the Ph_2PS ligands.

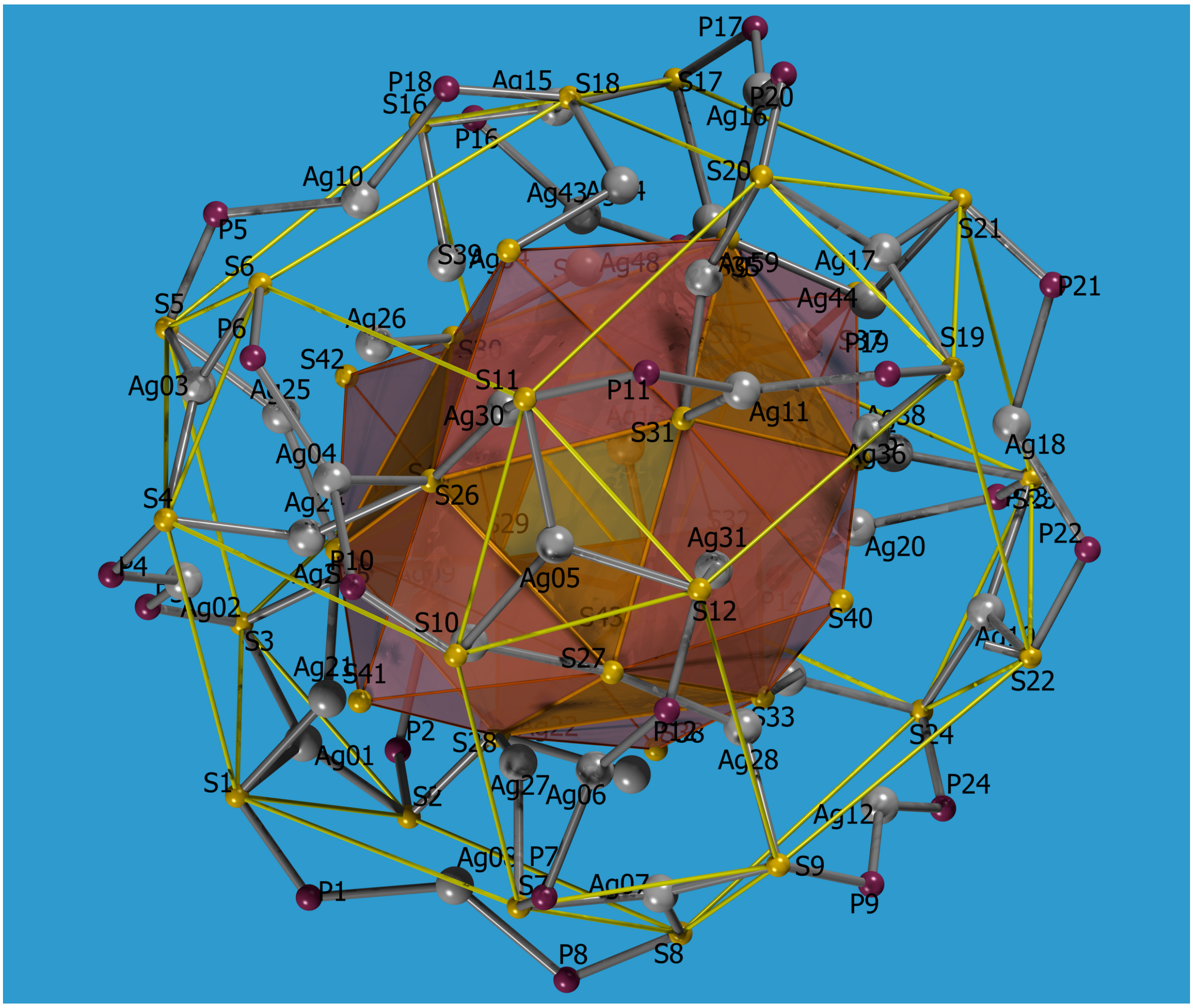

Figure S11. The arrangement of the 20 sulphide ions in **1**.

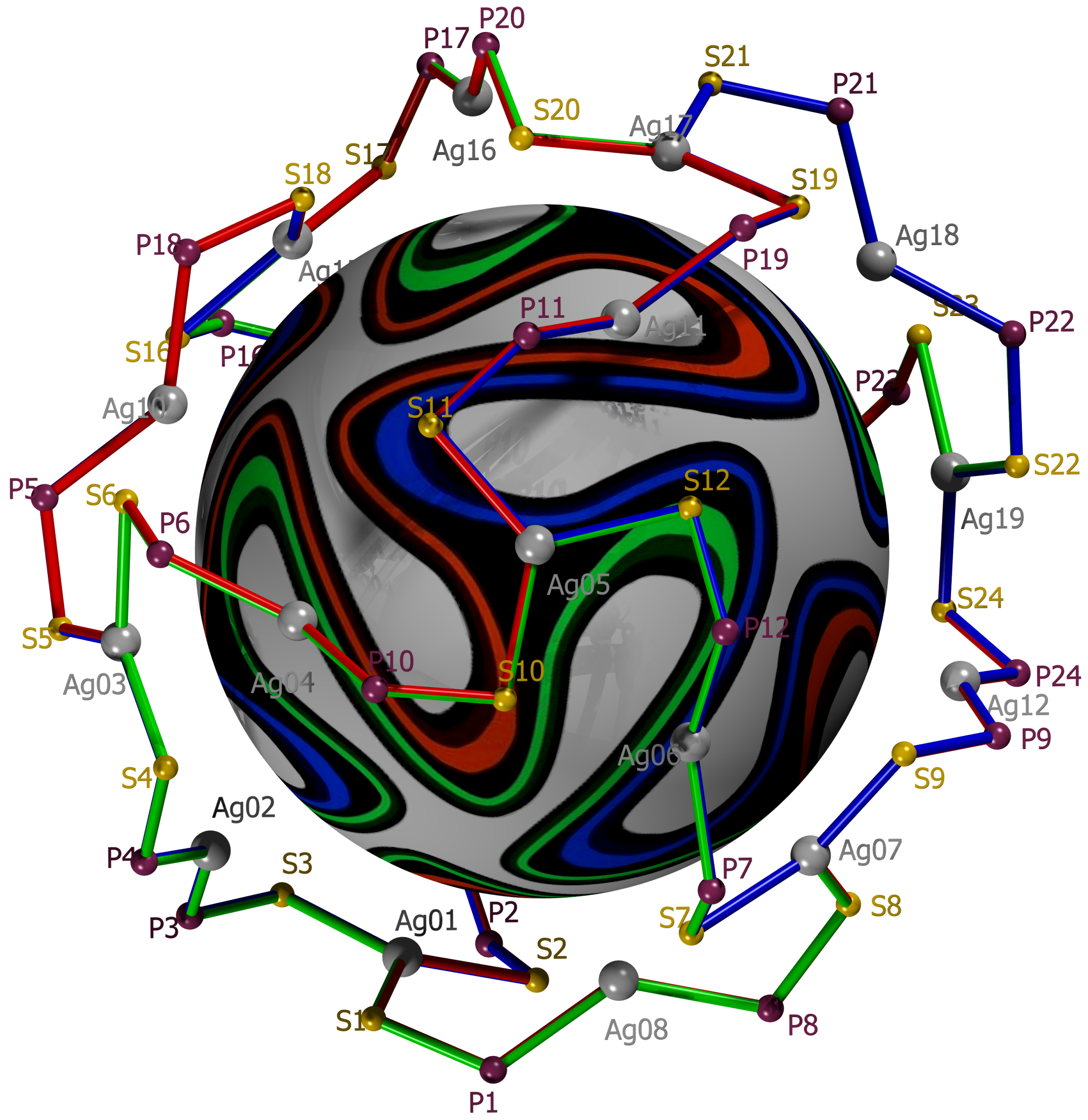

Figure S12. The motif formed by the P and S ligand atoms and the outer 20 Ag atoms in cluster **1** and the FIFA championship soccer ball, Brazuca, Adidas, Brazil 2014.

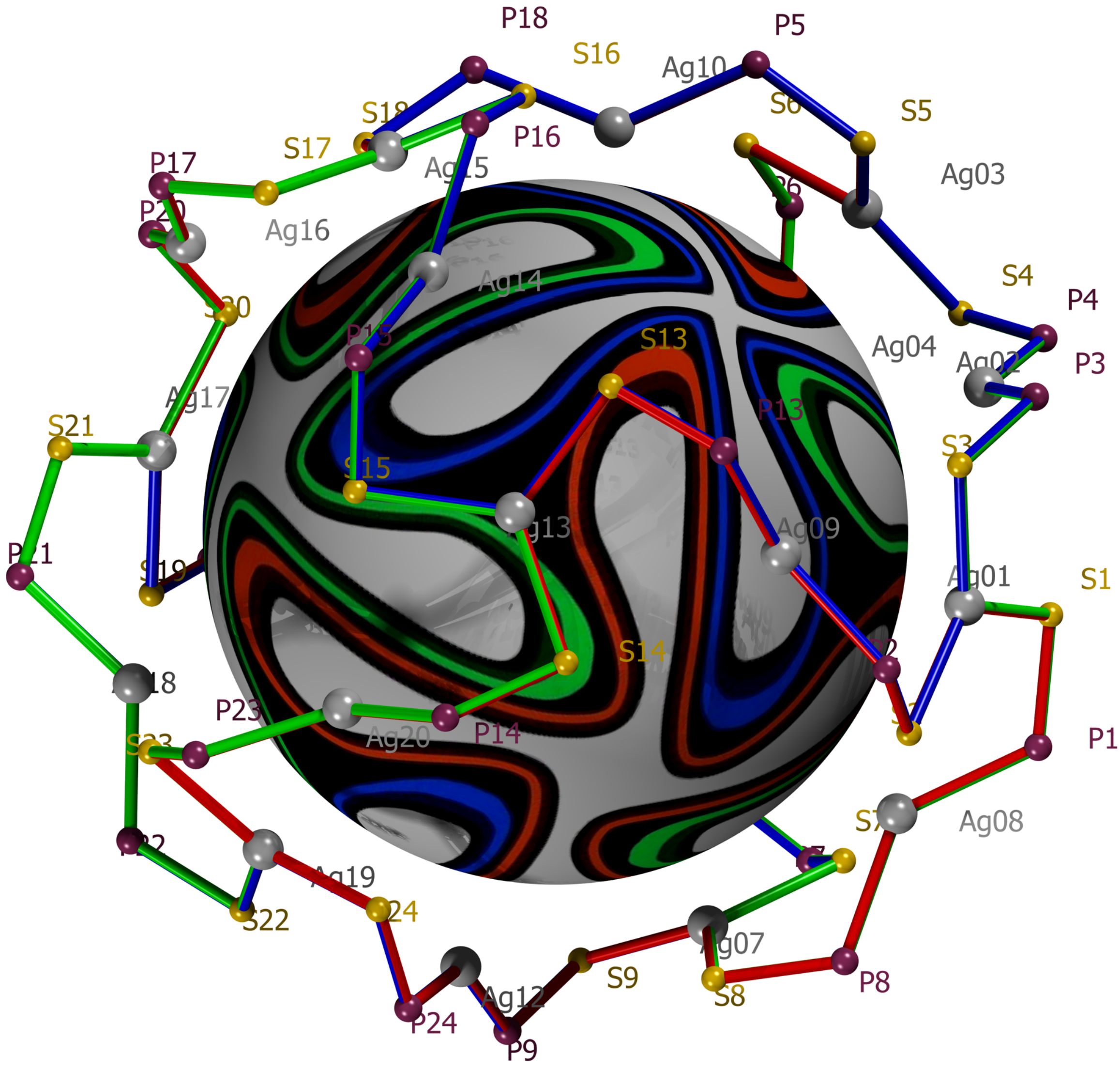

Figure S13. The motif formed by the P and S ligand atoms and the outer 20 Ag atoms in cluster **1** and the FIFA championship soccer ball, Brazuca, Adidas, Brazil 2014.

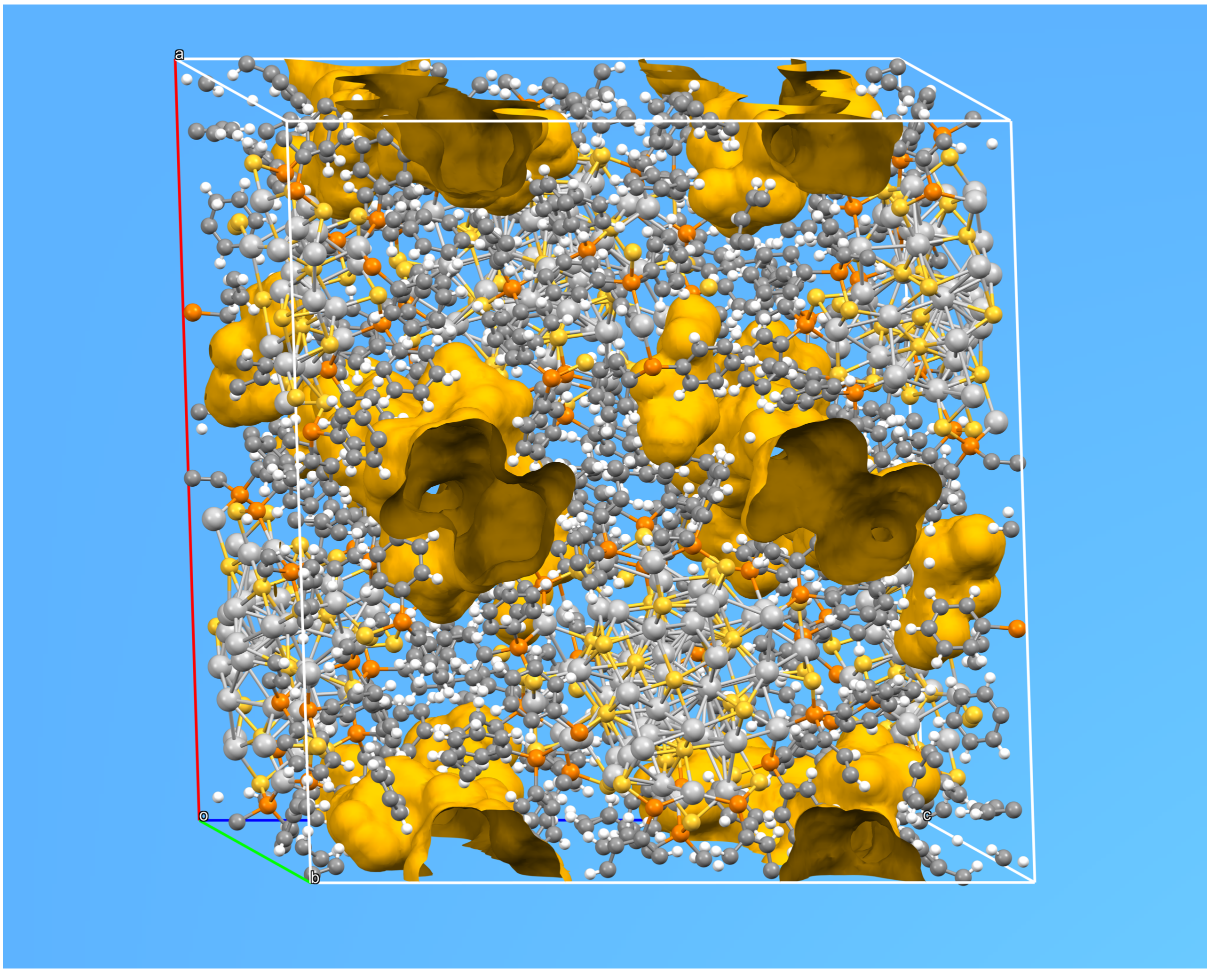

Figure S14. Void space within the unit cell of **1**, indicated by the brown surface. The total solvent accessible area (1.2 Å sphere) is 4635 Å³ (12.9 % of the unit cell volume).

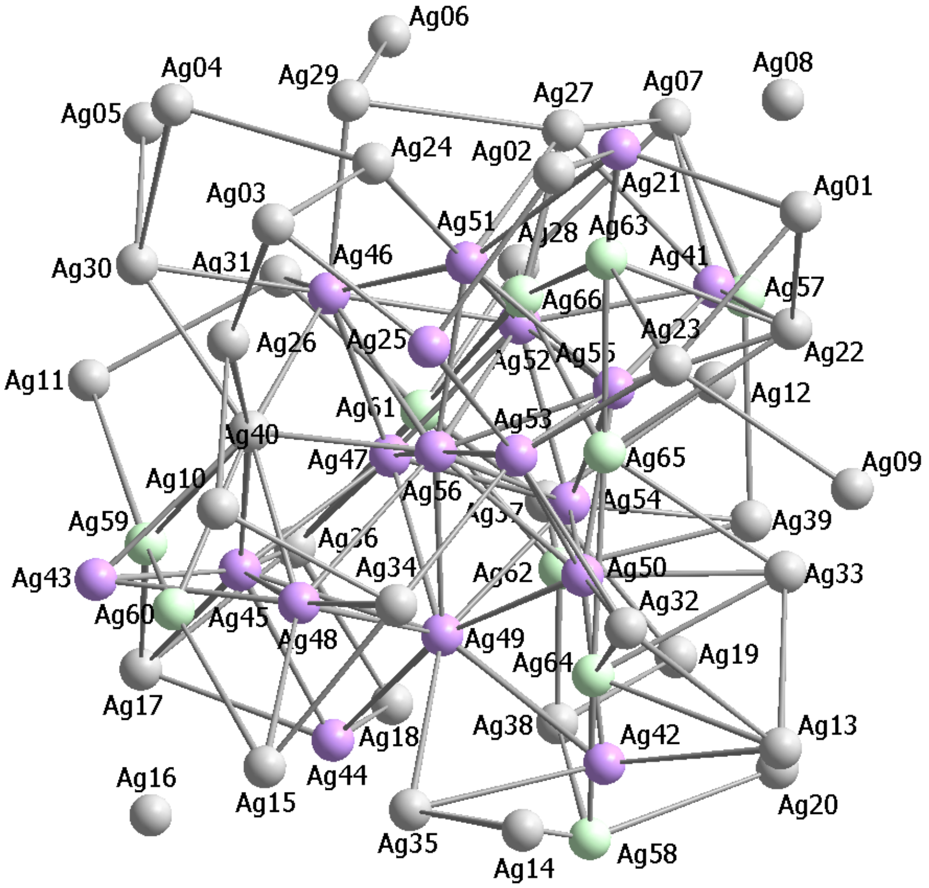

Figure S15. Disorder of the Ag atoms in **1**, with Ag occupancy of 1 in silver (main group), with occupancy between 0.5 and 1 in purple (Part 1) and with occupancy less than 0.5 in green (Part 2). The occupancy of the disordered Ag atoms was refined freely; no constraints nor restraints were applied. Ag–Ag bonds were only allowed between the Ag atoms of the main group and those of Part 1 and between the Ag atoms of the main group and those of Part 2.

Text S1. When redox reactions are involved in cluster synthesis the details are mostly unknown, only for synthesis involving purely anion-cation metathesis the exact stoichiometry can be presented. Approximate numbers were used; 1.2 should bring the number of Ag atoms from 44 to 53. 22 Ag(0) atoms must be oxidized to Ag(1) by formally 11 H_2S (formed from $Ph_2P(S)H$), which is thermodynamically feasible; the remaining Ag(1) ions will react via metathesis reactions with $Ph_2P(S)H$ and H_2S. Complexation of the SPS ligands will generate an additional driving force. In the Fenske syntheses $(TMS)_2$ is similarly generated from $(TMS)_2S$ as sulfur donor. Dihydrogen may have been consumed by reduction of dichloromethane solvent, but both reactions remained unnoticed. For a general scheme, see Fenske.

O. Fuhr, S. Dehnen and D. Fenske, Chalcogenide clusters of copper and silver from silylated chalcogenide sources, Chem. Soc. Rev., 2013, 42, 1871–1906 (reference 25).

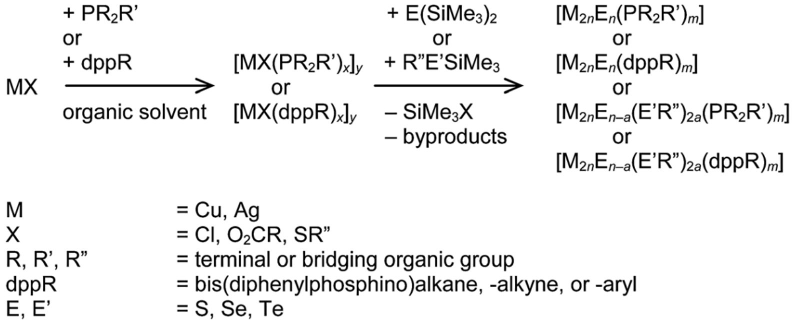

"General synthesis route for the formation of phosphane-ligated, chalcogen-bridged copper and silver clusters. Main byproducts are M_2E, EPR_2R0, $(Me_3Si)_2$, $(R00E)_2$."

Standard free energies of formation of two compounds:
Ag_2S: $\Delta F = -9.5$ kcal.mol^{-1}. https://doi.org/10.1021/ja01146a063

H_2S: $\Delta F = -7.9$ kcal.mol^{-1}. Stull, D. R.; Westrum, E. F.; and Sinke, G. C. *The Chemical Thermodynamics of Organic Compounds*, 882 p. ISBN-10 9780471834908, Wiley, New York, 1969.
