# Supplementary material for: A Silver Sulfide Cluster with Exterior Diphenylphosphinothioito Ligands Exhibiting a Triskele Motif
Source: ACS Omega. 2025 Dec 2;10(49):60572–8. doi: 10.1021/acsomega.5c08304 (PMC12713494; doi:10.1021/acsomega.5c08304)

checkCIF/PLATON report

Structure factors have been supplied for datablock(s) RB_UFRGS_HS_GM79_100K_Mo

THIS REPORT IS FOR GUIDANCE ONLY. IF USED AS PART OF A REVIEW PROCEDURE FOR PUBLICATION, IT SHOULD NOT REPLACE THE EXPERTISE OF AN EXPERIENCED CRYSTALLOGRAPHIC REFEREE.

No syntax errors found.   CIF dictionary   Interpreting this report

Datablock: RB_UFRGS_HS_GM79_100K_Mo

Bond precision:	C-C = 0.0370 A		Wavelength=0.71073
Cell:	a=40.070(7)	b=23.150(4)	c=38.910(7)
	alpha=90	beta=93.916(4)	gamma=90
Temperature:	100 K		

	Calculated	Reported
Volume	36009(11)	36009(11)
Space group	P 2/c	P 1 2/c 1
Hall group	-P 2yc	-P 2yc
Moiety formula	C288 H240 Ag53 P24 S44 [+ solvent]	?
Sum formula	C288 H240 Ag53 P24 S44 [+ solvent]	C288 H240 Ag53 P24 S44
Mr	11570.97	11571.82
Dx,g cm-3	2.134	2.134
Z	4	4
Mu (mm-1)	3.210	3.210
F000	22090.7	22092.0
F000'	21936.44	
h,k,lmax	53,30,52	53,30,51
Nref	90134	89620
Tmin,Tmax	0.486,0.852	0.290,0.490
Tmin'	0.380	

Correction method= # Reported T Limits: Tmin=0.290 Tmax=0.490
AbsCorr = MULTI-SCAN

Data completeness= 0.994 Theta(max)= 28.370

R(reflections)= 0.1985(62425) wR2(reflections)= 0.4674(89620)

S = 1.088 Npar= 3251

The following ALERTS were generated. Each ALERT has the format
test-name_ALERT_alert-type_alert-level.
Click on the hyperlinks for more details of the test.

Alert level A

PLAT084_ALERT_3_A High wR2 Value (i.e. > 0.25) .... 0.47 Report

Author Response: The structure was modelled as best possible, given the extreme complexity of the structure. The outside atoms of the cluster refine acceptably without instability. The very inside atoms of the cluster are highly disordered and were modelled to account for all residual electron density. Much effort was made to attribute the disorderd Ag atoms in the core of the structure with many trial strucures being refined. The least complicated structure which revealed the best structure was ultimately chosen.

PLAT213_ALERT_2_A Atom C10G has ADP max/min Ratio ..... 6.7 prolat

Author Response: The C atoms of the phenyl groups were modelled anisotropically but constrained with RIGU, SIMU and ISOR instructions. Of the 144 C atoms, just 11 were prolate. The C atoms contribure just 864 e^-^ of a total of 4504 e^-^ of the cluster. Additionally, atoms Ag28 and S28 are slightly prolate. These atoms were checked and appear to be modelled correctly.

PLAT411_ALERT_2_A Short Inter H...H Contact H4E ..H4E . 1.29 Ang.
1-x,y,1/2-z = 2_655 Check

Author Response: The close contact is between two H atoms of symmetry related phenyl groups. These is no doubt of their positions.

PLAT971_ALERT_2_A Check Calcd Resid. Dens. 1.24Ang From Ag21 8.58 eA-3
PLAT971_ALERT_2_A Check Calcd Resid. Dens. 1.00Ang From Ag21 8.55 eA-3
PLAT971_ALERT_2_A Check Calcd Resid. Dens. 1.38Ang From Ag49 8.36 eA-3
PLAT971_ALERT_2_A Check Calcd Resid. Dens. 1.07Ang From Ag63 6.78 eA-3
PLAT971_ALERT_2_A Check Calcd Resid. Dens. 0.93Ang From Ag32 6.49 eA-3
PLAT971_ALERT_2_A Check Calcd Resid. Dens. 1.02Ang From Ag36 6.13 eA-3
PLAT971_ALERT_2_A Check Calcd Resid. Dens. 1.07Ang From Ag48 5.98 eA-3
PLAT971_ALERT_2_A Check Calcd Resid. Dens. 0.99Ang From Ag35 5.95 eA-3

```
PLAT971_ALERT_2_A Check Calcd Resid. Dens.  1.93Ang From Ag50           5.89 eA-3
PLAT971_ALERT_2_A Check Calcd Resid. Dens.  1.00Ang From Ag25           5.57 eA-3
PLAT971_ALERT_2_A Check Calcd Resid. Dens.  1.18Ang From Ag46           5.29 eA-3
PLAT971_ALERT_2_A Check Calcd Resid. Dens.  0.94Ang From Ag53           5.01 eA-3
PLAT971_ALERT_2_A Check Calcd Resid. Dens.  1.17Ang From S18            4.88 eA-3
PLAT971_ALERT_2_A Check Calcd Resid. Dens.  0.91Ang From Ag50           4.77 eA-3
PLAT971_ALERT_2_A Check Calcd Resid. Dens.  0.88Ang From Ag56           4.60 eA-3
PLAT971_ALERT_2_A Check Calcd Resid. Dens.  0.95Ang From Ag48           4.59 eA-3
PLAT971_ALERT_2_A Check Calcd Resid. Dens.  0.52Ang From Ag38           4.56 eA-3
PLAT971_ALERT_2_A Check Calcd Resid. Dens.  0.88Ang From Ag10           4.50 eA-3
PLAT971_ALERT_2_A Check Calcd Resid. Dens.  0.85Ang From Ag34           4.43 eA-3
PLAT971_ALERT_2_A Check Calcd Resid. Dens.  0.87Ang From C18G           4.23 eA-3
PLAT971_ALERT_2_A Check Calcd Resid. Dens.  1.03Ang From Ag52           4.17 eA-3
PLAT971_ALERT_2_A Check Calcd Resid. Dens.  0.94Ang From Ag49           3.92 eA-3
PLAT971_ALERT_2_A Check Calcd Resid. Dens.  0.90Ang From Ag23           3.88 eA-3
PLAT971_ALERT_2_A Check Calcd Resid. Dens.  1.08Ang From Ag36           3.87 eA-3
PLAT971_ALERT_2_A Check Calcd Resid. Dens.  1.35Ang From S44            3.86 eA-3
PLAT972_ALERT_2_A Check Calcd Resid. Dens.  1.10Ang From Ag38          -5.05 eA-3
PLAT972_ALERT_2_A Check Calcd Resid. Dens.  0.74Ang From Ag10          -4.12 eA-3
PLAT972_ALERT_2_A Check Calcd Resid. Dens.  1.14Ang From Ag58          -4.10 eA-3
PLAT972_ALERT_2_A Check Calcd Resid. Dens.  0.84Ang From Ag26          -3.99 eA-3
PLAT972_ALERT_2_A Check Calcd Resid. Dens.  0.93Ang From Ag32          -3.69 eA-3
PLAT974_ALERT_2_A Check Calcd Negative Resid. Density on      Ag38      -3.67 eA-3
```

Author Response: This Ag atom was refined with partial occupancy but gave very close to unity so was left at that value.

Alert level B

```
PLAT082_ALERT_2_B High R1 Value ................................       0.20 Report
```

Author Response: The structure was modelled as best possible, given the extreme complexity of the structure. The outside atoms of the cluster refine acceptably without instability. The very inside atoms of the cluster are highly disordered and were modelled to account for all residual electron density. Much effort was made to attribute the disorderd Ag atoms in the core of the structure with many trial strucures being refined. The least complicated structure which revealed the best structure was ultimately chosen. The final structure refined well.

```
PLAT097_ALERT_2_B Large Reported Max.  (Positive) Residual Density       8.75 eA-3
```

Author Response: This large density is about 1.22 \%A from Ag21 and occurs in a region, the center of the cluster, dominated by disordered Ag atoms. Much effort was made to attribute the disorderd Ag atoms in the core of the structure with many trial strucures being refined. The least complicated structure which revealed the best structure was ultimately chosen.

```
PLAT213_ALERT_2_B Atom C4H             has ADP max/min Ratio .....        4.3 prolat
```

Author Response: The C atoms of the phenyl groups were modelled anisotropically but constrained with RIGU, SIMU and ISOR instructions. Of the 144 C atoms, just 11 were prolate. The C atoms contribure just 864 e^-^ of a total of 4504 e^-^ of the cluster. Additionally, atoms Ag28 and S28 are slightly prolate. These atoms were checked and appear to be modelled correctly.

```
PLAT213_ALERT_2_B Atom C9A             has ADP max/min Ratio .....        4.1 prolat
```

Author Response: The C atoms of the phenyl groups were modelled anisotropically but constrained with RIGU, SIMU and ISOR instructions. Of the 144 C atoms, just 11 were prolate. The C atoms contribure just 864 e^-^ of a total of 4504 e^-^ of the cluster. Additionally, atoms Ag28 and S28 are slightly prolate. These atoms were checked and appear to be modelled correctly.

```
PLAT213_ALERT_2_B Atom C13I            has ADP max/min Ratio .....        4.1 prolat
```

Author Response: The C atoms of the phenyl groups were modelled anisotropically but constrained with RIGU, SIMU and ISOR instructions. Of the 144 C atoms, just 11 were prolate. The C atoms contribure just 864 e^-^ of a total of 4504 e^-^ of the cluster. Additionally, atoms Ag28 and S28 are slightly prolate. These atoms were checked and appear to be modelled correctly.

```
PLAT213_ALERT_2_B Atom C14G            has ADP max/min Ratio .....        4.6 prolat
```

Author Response: The C atoms of the phenyl groups were modelled anisotropically but constrained with RIGU, SIMU and ISOR instructions. Of the 144 C atoms, just 11 were prolate. The C atoms contribure just 864 e^-^ of a total of 4504 e^-^ of the cluster. Additionally, atoms Ag28 and S28 are slightly prolate. These atoms were checked and appear to be modelled correctly.

```
PLAT220_ALERT_2_B NonSolvent   Resd 1  C   Ueq(max)/Ueq(min) Range        7.3 Ratio
```

Author Response: In the case for a C atom, this is C10G. The C atoms of the phenyl groups were modelled anisotropically but constrained with RIGU, SIMU and ISOR instructions. Of the 144 C atoms, just 11 were prolate. The C atoms contribure just 864 e^-^ of a total of 4504 e^-^ of the cluster. In the case for a Ag atom, this is Ag38 which shows a large negative difference peak close by. In the case for a S atom, this is S44 which is located within the disordered core of the cluster.

```
PLAT241_ALERT_2_B High   'MainMol' Ueq as Compared to Neighbors of       Ag38 Check
```

Author Response: The atoms Ag38, S44, Ag23, Ag28, Ag29, Ag31, Ag32, Ag33, Ag36, S8, S28, S30, S43, C2G, C2J, C3G, C4A, C4G, C12K, C12L, C21K e C24C have the high 'MainMol' Ue as compared to neighbors. The final refinements of the Ag atoms were with fixed unity occupations, but were each checked using variable occupancies which for each refined to close to unity. Ths S and C have to apparent disorder and are flagged since their neighbors have low 'MainMaol' Ueq values. Much effort was made to attribute the disorderd Ag atoms in the core of the structure with many trial strucures being refined. The least complicated structure which revealed the best structure was ultimately chosen.

```
PLAT241_ALERT_2_B High   'MainMol' Ueq as Compared to Neighbors of        S44 Check
```

Author Response: The atoms Ag38, S44, Ag23, Ag28, Ag29, Ag31, Ag32, Ag33, Ag36, S8, S28, S30, S43, C2G, C2J, C3G, C4A, C4G, C12K, C12L, C21K e C24C have the high 'MainMol' Ue as compared to neighbors. The final refinements of the Ag atoms were with fixed unity occupations, but were each checked using variable occupancies which for each refined to close to unity. Ths S and C have to apparent disorder and are flagged since their neighbors have low 'MainMaol' Ueq values. Much effort was made to attribute the disorderd Ag atoms in the core of the structure with many trial strucures being refined. The least complicated structure which revealed the best structure was ultimately chosen.

```
PLAT342_ALERT_3_B Low Bond Precision on  C-C Bonds ..............    0.03705 Ang.
```

Author Response: The structure is extremely complex with many heavy atoms (53 Ag atoms), thus the contributions of the C atoms in the structure factors is quite small and leads to larger errors in their determinations. All phenyl groups were refined using AFIX 66 with the standard C-C distance of 1.39 \%A.

```
PLAT910_ALERT_3_B Missing FCF Reflection(s) Below Theta(Min)[Deg]=       2.12 Note
                0  1  0,   0  2  0,   1  0  0,   1  1  0,   1  2  0,   2  0  0,
                2  1  0,   2  2  0,   3  0  0,   3  1  0,   4  0  0,  -3  1  1,
               -2  1  1,  -2  2  1,  -1  1  1,  -1  2  1,   0  1  1,   0  2  1,
                1  1  1,   1  2  1,   2  1  1,   3  1  1,  -3  0  2,  -3  1  2,
                  (   19 More Missing: see the .ckf listing file)
```

Author Response: All but 10 of these reflections at below 2.12 \% and are occluded by the beam stop. The remaining 10 were measured but were eliminated by SAINT for exceeding the image queue.

```
PLAT934_ALERT_3_B Number of (Iobs-Icalc)/Sigma(W) > 10 Outliers ..          2 Check
                8   6   5,    0   1  14,
```

Author Response: These reflections may have suffered severe absorption effects.

```
PLAT972_ALERT_2_B Check Calcd Resid. Dens.  1.57Ang From C24B          -3.46 eA-3
PLAT972_ALERT_2_B Check Calcd Resid. Dens.  0.67Ang From Ag35          -3.31 eA-3
PLAT972_ALERT_2_B Check Calcd Resid. Dens.  0.73Ang From Ag40          -3.27 eA-3
PLAT972_ALERT_2_B Check Calcd Resid. Dens.  0.69Ang From Ag36          -3.22 eA-3
PLAT972_ALERT_2_B Check Calcd Resid. Dens.  0.95Ang From C9E           -3.20 eA-3
PLAT972_ALERT_2_B Check Calcd Resid. Dens.  0.79Ang From Ag23          -3.14 eA-3
PLAT972_ALERT_2_B Check Calcd Resid. Dens.  1.40Ang From Ag56          -3.13 eA-3
PLAT972_ALERT_2_B Check Calcd Resid. Dens.  1.27Ang From S24           -3.10 eA-3
PLAT972_ALERT_2_B Check Calcd Resid. Dens.  0.84Ang From Ag34          -3.08 eA-3
PLAT972_ALERT_2_B Check Calcd Resid. Dens.  1.60Ang From C14B          -3.02 eA-3
PLAT972_ALERT_2_B Check Calcd Resid. Dens.  0.78Ang From Ag28          -3.00 eA-3
PLAT972_ALERT_2_B Check Calcd Resid. Dens.  0.98Ang From Ag54          -2.98 eA-3
PLAT972_ALERT_2_B Check Calcd Resid. Dens.  0.47Ang From Ag36          -2.96 eA-3
PLAT972_ALERT_2_B Check Calcd Resid. Dens.  1.09Ang From S24           -2.95 eA-3
PLAT972_ALERT_2_B Check Calcd Resid. Dens.  0.86Ang From C22B          -2.92 eA-3
PLAT972_ALERT_2_B Check Calcd Resid. Dens.  1.45Ang From S15           -2.92 eA-3
PLAT972_ALERT_2_B Check Calcd Resid. Dens.  1.34Ang From C21H          -2.86 eA-3
PLAT972_ALERT_2_B Check Calcd Resid. Dens.  0.60Ang From Ag17          -2.85 eA-3
PLAT972_ALERT_2_B Check Calcd Resid. Dens.  1.46Ang From C24E          -2.83 eA-3
PLAT977_ALERT_2_B Check Negative Difference Density on H18D      .      -1.80 eA-3
```

Author Response: Many H atoms had negative difference electron density. It might be due to the PLATON SQUEEZE routine.

```
PLAT977_ALERT_2_B Check Negative Difference Density on H24C      .      -1.79 eA-3
```

Author Response: Many H atoms had negative difference electron density. It might be due to the PLATON SQUEEZE routine.

Alert level C

```
DIFMN02_ALERT_2_C  The minimum difference density is < -0.1*ZMAX*0.75
            _refine_diff_density_min given =     -4.601
            Test value =     -3.525
```

Author Response: The largest negative difference density of -4.6 e/\%A occurs at 0.16 \%A from Ag38. Much effort was made to attribute the disorderd Ag atoms in the core of the structure with many trial strucures being refined. The least complicated structure which revealed the best structure was ultimately chosen.

```
DIFMN03_ALERT_1_C  The minimum difference density is < -0.1*ZMAX*0.75
            The relevant atom site should be identified.
```

Author Response: The largest negative difference density of -4.6 e \%A^-3^ occurs at 0.16 \%A from Ag38. Much effort was made to attribute the disorderd Ag atoms in core of the structure with many trial strucures being refined. The least complicated structure which revealed the best structure was ultimately chosen.

```
DIFMX02_ALERT_1_C  The maximum difference density is > 0.1*ZMAX*0.75
            The relevant atom site should be identified.
```

Author Response: The largest positive difference density of 8.75 e\%A^-3^ occurs at 1.22 \%A from Ag21. Much effort was made to attribute the disorderd Ag atoms in core of the structure with many trial strucures being refined. The least complicated structure which revealed the best structure was ultimately chosen.

```
RINTA01_ALERT_3_C  The value of Rint is greater than 0.12
            Rint given   0.166
```

Author Response: The R~int~ of the final refinement is 0.166. This is due in part to the high multiplicity of observations. 1024921 reflections were measured to a 2\t of 56.74 \% with just 89620 unique, giving an average multiplicity of 11.4. R~int~ always sums the differences, without a denominator for the number of difference pairs, thus the greater the multiplicity of observations, the higher the value of R~int~. It is also due in part to the slightly weak data; R~sigma~ = 0.0823. The Photon 100 detector has a limit of 60 s for exposure times; higher than this causes pixel damage.

```
PLAT020_ALERT_3_C The Value of Rint is Greater Than 0.12 .........      0.166 Report
```

Author Response: The R~int~ of the final refinement is 0.166. This is due in part to the high multiplicity of observations. 1024921 reflections were measured to a 2\t of 56.74 \% with just 89620 unique, giving an average multiplicity of 11.4. R~int~ always sums the differences, without a denominator for the number of difference pairs, thus the greater the multiplicity of observations, the higher the value of R~int~. It is also due in part to the slightly weak data; R~sigma~ = 0.0823. The Photon 100 detector as a limit of 60 s for exposure times; higher than this causes pixel damage.

```
PLAT048_ALERT_1_C MoietyFormula Not Given (or Incomplete) ........     Please Check
PLAT098_ALERT_2_C Large Reported Min.  (Negative) Residual Density      -4.60 eA-3
```

Author Response: The largest negative difference density of -4.6 e \%A^-3^ occurs at 0.16 \%A from Ag38, which was refined with partial occupancy but gave very close to unity so was left at that value.

```
PLAT213_ALERT_2_C Atom Ag38            has ADP max/min Ratio .....        3.1 prolat
```

Author Response: The C atoms of the phenyl groups were modelled anisotropically but constrained with RIGU, SIMU and ISOR instructions. Of the 144 C atoms, just 11 were prolate. The C atoms contribure just 864 e^-^ of a total of 4504 e^-^ of the cluster. Additionally, atoms Ag28 and S28 are slightly prolate. These atoms were checked and appear to be modelled correctly.

```
PLAT213_ALERT_2_C Atom S28             has ADP max/min Ratio .....        3.1 prolat
```

Author Response: The C atoms of the phenyl groups were modelled anisotropically but constrained with RIGU, SIMU and ISOR instructions. Of the 144 C atoms, just 11 were prolate. The C atoms contribure just 864 e^-^ of a total of 4504 e^-^ of the cluster. Additionally, atoms Ag28 and S28 are slightly prolate. These atoms were checked and appear to be modelled correctly.

```
PLAT213_ALERT_2_C Atom C3A             has ADP max/min Ratio .....        3.2 prolat
```

Author Response: The C atoms of the phenyl groups were modelled anisotropically but constrained with RIGU, SIMU and ISOR instructions. Of the 144 C atoms, just 11 were prolate. The C atoms contribure just 864 e^-^ of a total of 4504 e^-^ of the cluster. Additionally, atoms Ag28 and S28 are slightly prolate. These atoms were checked and appear to be modelled correctly.

```
PLAT213_ALERT_2_C Atom C4I             has ADP max/min Ratio .....        3.6 oblate
```

Author Response: The C atoms of the phenyl groups were modelled anisotropically but constrained with RIGU, SIMU and ISOR instructions. Of the 144 C atoms, just 11 were prolate. The C atoms contribure just 864 e^-^ of a total of 4504 e^-^ of the cluster. Additionally, atoms Ag28 and S28 are slightly prolate. These atoms were checked and appear to be modelled correctly.

```
PLAT213_ALERT_2_C Atom C4J             has ADP max/min Ratio .....        3.5 oblate
```

Author Response: The C atoms of the phenyl groups were modelled anisotropically but constrained with RIGU, SIMU and ISOR instructions. Of the 144 C atoms, just 11 were prolate. The C atoms contribure just 864 e^-^ of a total of 4504 e^-^ of the cluster. Additionally, atoms Ag28 and S28 are slightly prolate. These atoms were checked and appear to be modelled correctly.

PLAT213_ALERT_2_C Atom C6B has ADP max/min Ratio ..... 3.5 prolat

Author Response: The C atoms of the phenyl groups were modelled anisotropically but constrained with RIGU, SIMU and ISOR instructions. Of the 144 C atoms, just 11 were prolate. The C atoms contribure just 864 e^-^ of a total of 4504 e^-^ of the cluster. Additionally, atoms Ag28 and S28 are slightly prolate. These atoms were checked and appear to be modelled correctly.

PLAT213_ALERT_2_C Atom C10H has ADP max/min Ratio ..... 3.6 prolat

Author Response: The C atoms of the phenyl groups were modelled anisotropically but constrained with RIGU, SIMU and ISOR instructions. Of the 144 C atoms, just 11 were prolate. The C atoms contribure just 864 e^-^ of a total of 4504 e^-^ of the cluster. Additionally, atoms Ag28 and S28 are slightly prolate. These atoms were checked and appear to be modelled correctly.

PLAT213_ALERT_2_C Atom C10L has ADP max/min Ratio ..... 3.1 prolat

Author Response: The C atoms of the phenyl groups were modelled anisotropically but constrained with RIGU, SIMU and ISOR instructions. Of the 144 C atoms, just 11 were prolate. The C atoms contribure just 864 e^-^ of a total of 4504 e^-^ of the cluster. Additionally, atoms Ag28 and S28 are slightly prolate. These atoms were checked and appear to be modelled correctly.

PLAT213_ALERT_2_C Atom C14K has ADP max/min Ratio ..... 4.0 oblate

Author Response: The C atoms of the phenyl groups were modelled anisotropically but constrained with RIGU, SIMU and ISOR instructions. Of the 144 C atoms, just 11 were prolate. The C atoms contribure just 864 e^-^ of a total of 4504 e^-^ of the cluster. Additionally, atoms Ag28 and S28 are slightly prolate. These atoms were checked and appear to be modelled correctly.

PLAT220_ALERT_2_C NonSolvent Resd 1 Ag Ueq(max)/Ueq(min) Range 5.0 Ratio

Author Response: In the case for a C atom, this is C10G. The C atoms of the phenyl groups were modelled anisotropically but constrained with RIGU, SIMU and ISOR instructions. Of the 144 C atoms, just 11 were prolate. The C atoms contribure just 864 e^-^ of a total of 4504 e^-^ of the cluster. In the case for a Ag atom, this is Ag38 which shows a large negative difference peak close by. In the case for a S atom, this is S44 which is located within the disordered core of the cluster.

```
PLAT220_ALERT_2_C NonSolvent   Resd 1  S   Ueq(max)/Ueq(min) Range        3.9 Ratio
```

Author Response: In the case for a C atom, this is C10G. The C atoms of the phenyl groups were modelled anisotropically but constrained with RIGU, SIMU and ISOR instructions. Of the 144 C atoms, just 11 were prolate. The C atoms contribure just 864 e^-^ of a total of 4504 e^-^ of the cluster. In the case for a Ag atom, this is Ag38 which shows a large negative difference peak close by. In the case for a S atom, this is S44 which is located within the disordered core of the cluster.

```
PLAT222_ALERT_3_C NonSolvent Resd 1  H   Uiso(max)/Uiso(min) Range        6.7 Ratio
```

Author Response: This is due to the large U(iso) of the very prolate C atoms.

```
PLAT241_ALERT_2_C High   'MainMol' Ueq as Compared to Neighbors of       Ag23 Check
```

Author Response: The atoms Ag38, S44, Ag23, Ag28, Ag29, Ag31, Ag32, Ag33, Ag36, S8, S28, S30, S43, C2G, C2J, C3G, C4A, C4G, C12K, C12L, C21K e C24C have the high 'MainMol' Ue as compared to neighbors. The final refinements of the Ag atoms were with fixed unity occupations, but were each checked using variable occupancies which for each refined to close to unity. Ths S and C have to apparent disorder and are flagged since their neighbors have low 'MainMaol' Ueq values. Much effort was made to attribute the disorderd Ag atoms in the core of the structure with many trial strucures being refined. The least complicated structure which revealed the best structure was ultimately chosen.

```
PLAT241_ALERT_2_C High   'MainMol' Ueq as Compared to Neighbors of       Ag28 Check
```

Author Response: The atoms Ag38, S44, Ag23, Ag28, Ag29, Ag31, Ag32, Ag33, Ag36, S8, S28, S30, S43, C2G, C2J, C3G, C4A, C4G, C12K, C12L, C21K e C24C have the high 'MainMol' Ue as compared to neighbors. The final refinements of the Ag atoms were with fixed unity occupations, but were each checked using variable occupancies which for each refined to close to unity. Ths S and C have to apparent disorder and are flagged since their neighbors have low 'MainMaol' Ueq values. Much effort was made to attribute the disorderd Ag atoms in the core of the structure with many trial strucures being refined. The least complicated structure which revealed the best structure was ultimately chosen.

```
PLAT241_ALERT_2_C High   'MainMol' Ueq as Compared to Neighbors of       Ag29 Check
```

Author Response: The atoms Ag38, S44, Ag23, Ag28, Ag29, Ag31, Ag32, Ag33, Ag36, S8, S28, S30, S43, C2G, C2J, C3G, C4A, C4G, C12K, C12L, C21K e C24C have the high 'MainMol' Ue as compared to neighbors. The final refinements of the Ag atoms were with fixed unity occupations, but were each checked using variable occupancies which for each refined to close to unity. Ths S and C have to apparent disorder and are flagged since their neighbors have low 'MainMaol' Ueq values. Much effort was made to attribute the disorderd Ag atoms in the core of the structure with many trial strucures being refined. The least complicated structure which revealed the best structure was ultimately chosen.

```
PLAT241_ALERT_2_C High   'MainMol' Ueq as Compared to Neighbors of       Ag31 Check
```

Author Response: The atoms Ag38, S44, Ag23, Ag28, Ag29, Ag31, Ag32, Ag33, Ag36, S8, S28, S30, S43, C2G, C2J, C3G, C4A, C4G, C12K, C12L, C21K e C24C have the high 'MainMol' Ue as compared to neighbors. The final refinements of the Ag atoms were with fixed unity occupations, but were each checked using variable occupancies which for each refined to close to unity. Ths S and C have to apparent disorder and are flagged since their neighbors have low 'MainMaol' Ueq values. Much effort was made to attribute the disorderd Ag atoms in the core of the structure with many trial strucures being refined. The least complicated structure which revealed the best structure was ultimately chosen.

```
PLAT241_ALERT_2_C High   'MainMol' Ueq as Compared to Neighbors of       Ag32 Check
```

Author Response: The atoms Ag38, S44, Ag23, Ag28, Ag29, Ag31, Ag32, Ag33, Ag36, S8, S28, S30, S43, C2G, C2J, C3G, C4A, C4G, C12K, C12L, C21K e C24C have the high 'MainMol' Ue as compared to neighbors. The final refinements of the Ag atoms were with fixed unity occupations, but were each checked using variable occupancies which for each refined to close to unity. Ths S and C have to apparent disorder and are flagged since their neighbors have low 'MainMaol' Ueq values. Much effort was made to attribute the disorderd Ag atoms in the core of the structure with many trial strucures being refined. The least complicated structure which revealed the best structure was ultimately chosen.

```
PLAT241_ALERT_2_C High   'MainMol' Ueq as Compared to Neighbors of       Ag33 Check
```

Author Response: The atoms Ag38, S44, Ag23, Ag28, Ag29, Ag31, Ag32, Ag33, Ag36, S8, S28, S30, S43, C2G, C2J, C3G, C4A, C4G, C12K, C12L, C21K e C24C have the high 'MainMol' Ue as compared to neighbors. The final refinements of the Ag atoms were with fixed unity occupations, but were each checked using variable occupancies which for each refined to close to unity. Ths S and C have to apparent disorder and are flagged since their neighbors have low 'MainMaol' Ueq values. Much effort was made to attribute the disorderd Ag atoms in the core of the structure with many trial strucures being refined. The least complicated structure which revealed the best structure was ultimately chosen.

PLAT241_ALERT_2_C High 'MainMol' Ueq as Compared to Neighbors of Ag36 Check

Author Response: The atoms Ag38, S44, Ag23, Ag28, Ag29, Ag31, Ag32, Ag33, Ag36, S8, S28, S30, S43, C2G, C2J, C3G, C4A, C4G, C12K, C12L, C21K e C24C have the high 'MainMol' Ue as compared to neighbors. The final refinements of the Ag atoms were with fixed unity occupations, but were each checked using variable occupancies which for each refined to close to unity. Ths S and C have to apparent disorder and are flagged since their neighbors have low 'MainMaol' Ueq values. Much effort was made to attribute the disorderd Ag atoms in the core of the structure with many trial strucures being refined. The least complicated structure which revealed the best structure was ultimately chosen.

PLAT241_ALERT_2_C High 'MainMol' Ueq as Compared to Neighbors of S8 Check

Author Response: The atoms Ag38, S44, Ag23, Ag28, Ag29, Ag31, Ag32, Ag33, Ag36, S8, S28, S30, S43, C2G, C2J, C3G, C4A, C4G, C12K, C12L, C21K e C24C have the high 'MainMol' Ue as compared to neighbors. The final refinements of the Ag atoms were with fixed unity occupations, but were each checked using variable occupancies which for each refined to close to unity. Ths S and C have to apparent disorder and are flagged since their neighbors have low 'MainMaol' Ueq values. Much effort was made to attribute the disorderd Ag atoms in the core of the structure with many trial strucures being refined. The least complicated structure which revealed the best structure was ultimately chosen.

PLAT241_ALERT_2_C High 'MainMol' Ueq as Compared to Neighbors of S28 Check

Author Response: The atoms Ag38, S44, Ag23, Ag28, Ag29, Ag31, Ag32, Ag33, Ag36, S8, S28, S30, S43, C2G, C2J, C3G, C4A, C4G, C12K, C12L, C21K e C24C have the high 'MainMol' Ue as compared to neighbors. The final refinements of the Ag atoms were with fixed unity occupations, but were each checked using variable occupancies which for each refined to close to unity. Ths S and C have to apparent disorder and are flagged since their neighbors have low 'MainMaol' Ueq values. Much effort was made to attribute the disorderd Ag atoms in the core of the structure with many trial strucures being refined. The least complicated structure which revealed the best structure was ultimately chosen.

PLAT241_ALERT_2_C High 'MainMol' Ueq as Compared to Neighbors of S30 Check

Author Response: The atoms Ag38, S44, Ag23, Ag28, Ag29, Ag31, Ag32, Ag33, Ag36, S8, S28, S30, S43, C2G, C2J, C3G, C4A, C4G, C12K, C12L, C21K e C24C have the high 'MainMol' Ue as compared to neighbors. The final refinements of the Ag atoms were with fixed unity occupations, but were each checked using variable occupancies which for each refined to close to unity. Ths S and C have to apparent disorder and are flagged since their neighbors have low 'MainMaol' Ueq values. Much effort was made to attribute the disorderd Ag atoms in the core of the structure with many trial strucures being refined. The least complicated structure which revealed the best structure was ultimately chosen.

PLAT241_ALERT_2_C High 'MainMol' Ueq as Compared to Neighbors of S43 Check

Author Response: The atoms Ag38, S44, Ag23, Ag28, Ag29, Ag31, Ag32, Ag33, Ag36, S8, S28, S30, S43, C2G, C2J, C3G, C4A, C4G, C12K, C12L, C21K e C24C have the high 'MainMol' Ue as compared to neighbors. The final refinements of the Ag atoms were with fixed unity occupations, but were each checked using variable occupancies which for each refined to close to unity. Ths S and C have to apparent disorder and are flagged since their neighbors have low 'MainMaol' Ueq values. Much effort was made to attribute the disorderd Ag atoms in the core of the structure with many trial strucures being refined. The least complicated structure which revealed the best structure was ultimately chosen.

PLAT241_ALERT_2_C High 'MainMol' Ueq as Compared to Neighbors of C2G Check

Author Response: The atoms Ag38, S44, Ag23, Ag28, Ag29, Ag31, Ag32, Ag33, Ag36, S8, S28, S30, S43, C2G, C2J, C3G, C4A, C4G, C12K, C12L, C21K e C24C have the high 'MainMol' Ue as compared to neighbors. The final refinements of the Ag atoms were with fixed unity occupations, but were each checked using variable occupancies which for each refined to close to unity. Ths S and C have to apparent disorder and are flagged since their neighbors have low 'MainMaol' Ueq values. Much effort was made to attribute the disorderd Ag atoms in the core of the structure with many trial strucures being refined. The least complicated structure which revealed the best structure was ultimately chosen.

```
PLAT241_ALERT_2_C High   'MainMol' Ueq as Compared to Neighbors of        C2J Check
```

Author Response: The atoms Ag38, S44, Ag23, Ag28, Ag29, Ag31, Ag32, Ag33, Ag36, S8, S28, S30, S43, C2G, C2J, C3G, C4A, C4G, C12K, C12L, C21K e C24C have the high 'MainMol' Ue as compared to neighbors. The final refinements of the Ag atoms were with fixed unity occupations, but were each checked using variable occupancies which for each refined to close to unity. Ths S and C have to apparent disorder and are flagged since their neighbors have low 'MainMaol' Ueq values. Much effort was made to attribute the disorderd Ag atoms in the core of the structure with many trial strucures being refined. The least complicated structure which revealed the best structure was ultimately chosen.

```
PLAT241_ALERT_2_C High   'MainMol' Ueq as Compared to Neighbors of        C3G Check
```

Author Response: The atoms Ag38, S44, Ag23, Ag28, Ag29, Ag31, Ag32, Ag33, Ag36, S8, S28, S30, S43, C2G, C2J, C3G, C4A, C4G, C12K, C12L, C21K e C24C have the high 'MainMol' Ue as compared to neighbors. The final refinements of the Ag atoms were with fixed unity occupations, but were each checked using variable occupancies which for each refined to close to unity. Ths S and C have to apparent disorder and are flagged since their neighbors have low 'MainMaol' Ueq values. Much effort was made to attribute the disorderd Ag atoms in the core of the structure with many trial strucures being refined. The least complicated structure which revealed the best structure was ultimately chosen.

```
PLAT241_ALERT_2_C High   'MainMol' Ueq as Compared to Neighbors of        C4A Check
```

Author Response: The atoms Ag38, S44, Ag23, Ag28, Ag29, Ag31, Ag32, Ag33, Ag36, S8, S28, S30, S43, C2G, C2J, C3G, C4A, C4G, C12K, C12L, C21K e C24C have the high 'MainMol' Ue as compared to neighbors. The final refinements of the Ag atoms were with fixed unity occupations, but were each checked using variable occupancies which for each refined to close to unity. Ths S and C have to apparent disorder and are flagged since their neighbors have low 'MainMaol' Ueq values. Much effort was made to attribute the disorderd Ag atoms in the core of the structure with many trial strucures being refined. The least complicated structure which revealed the best structure was ultimately chosen.

PLAT241_ALERT_2_C High 'MainMol' Ueq as Compared to Neighbors of C4G Check

Author Response: The atoms Ag38, S44, Ag23, Ag28, Ag29, Ag31, Ag32, Ag33, Ag36, S8, S28, S30, S43, C2G, C2J, C3G, C4A, C4G, C12K, C12L, C21K e C24C have the high 'MainMol' Ue as compared to neighbors. The final refinements of the Ag atoms were with fixed unity occupations, but were each checked using variable occupancies which for each refined to close to unity. Ths S and C have to apparent disorder and are flagged since their neighbors have low 'MainMaol' Ueq values. Much effort was made to attribute the disorderd Ag atoms in the core of the structure with many trial strucures being refined. The least complicated structure which revealed the best structure was ultimately chosen.

PLAT241_ALERT_2_C High 'MainMol' Ueq as Compared to Neighbors of C12K Check

Author Response: The atoms Ag38, S44, Ag23, Ag28, Ag29, Ag31, Ag32, Ag33, Ag36, S8, S28, S30, S43, C2G, C2J, C3G, C4A, C4G, C12K, C12L, C21K e C24C have the high 'MainMol' Ue as compared to neighbors. The final refinements of the Ag atoms were with fixed unity occupations, but were each checked using variable occupancies which for each refined to close to unity. Ths S and C have to apparent disorder and are flagged since their neighbors have low 'MainMaol' Ueq values. Much effort was made to attribute the disorderd Ag atoms in the core of the structure with many trial strucures being refined. The least complicated structure which revealed the best structure was ultimately chosen.

PLAT241_ALERT_2_C High 'MainMol' Ueq as Compared to Neighbors of C12L Check

Author Response: The atoms Ag38, S44, Ag23, Ag28, Ag29, Ag31, Ag32, Ag33, Ag36, S8, S28, S30, S43, C2G, C2J, C3G, C4A, C4G, C12K, C12L, C21K e C24C have the high 'MainMol' Ue as compared to neighbors. The final refinements of the Ag atoms were with fixed unity occupations, but were each checked using variable occupancies which for each refined to close to unity. Ths S and C have to apparent disorder and are flagged since their neighbors have low 'MainMaol' Ueq values. Much effort was made to attribute the disorderd Ag atoms in the core of the structure with many trial strucures being refined. The least complicated structure which revealed the best structure was ultimately chosen.

PLAT241_ALERT_2_C High 'MainMol' Ueq as Compared to Neighbors of C21K Check

Author Response: The atoms Ag38, S44, Ag23, Ag28, Ag29, Ag31, Ag32, Ag33, Ag36, S8, S28, S30, S43, C2G, C2J, C3G, C4A, C4G, C12K, C12L, C21K e C24C have the high 'MainMol' Ue as compared to neighbors. The final refinements of the Ag atoms were with fixed unity occupations, but were each checked using variable occupancies which for each refined to close to unity. Ths S and C have to apparent disorder and are flagged since their neighbors have low 'MainMaol' Ueq values. Much effort was made to attribute the disorderd Ag atoms in the core of the structure with many trial strucures being refined. The least complicated structure which revealed the best structure was ultimately chosen.

PLAT241_ALERT_2_C High 'MainMol' Ueq as Compared to Neighbors of C24C Check

Author Response: The atoms Ag38, S44, Ag23, Ag28, Ag29, Ag31, Ag32, Ag33, Ag36, S8, S28, S30, S43, C2G, C2J, C3G, C4A, C4G, C12K, C12L, C21K e C24C have the high 'MainMol' Ue as compared to neighbors. The final refinements of the Ag atoms were with fixed unity occupations, but were each checked using variable occupancies which for each refined to close to unity. Ths S and C have to apparent disorder and are flagged since their neighbors have low 'MainMaol' Ueq values. Much effort was made to attribute the disorderd Ag atoms in the core of the structure with many trial strucures being refined. The least complicated structure which revealed the best structure was ultimately chosen.

PLAT242_ALERT_2_C Low 'MainMol' Ueq as Compared to Neighbors of Ag07 Check

Author Response: The Ag07, Ag19, S23, S25, S27, S36, S37, S38, S40, S42, P2, P3, P4, P6, P8, P10, P20, C12I and C15J show no disorder and cannot be elements different to those attributed. These are close to atoms with large apparent thermal motions.

```
PLAT242_ALERT_2_C Low    'MainMol' Ueq as Compared to Neighbors of       Ag19 Check
```

Author Response: The Ag07, Ag19, S23, S25, S27, S36, S37, S38, S40, S42, P2, P3, P4, P6, P8, P10, P20, C12I and C15J show no disorder and cannot be elements different to those attributed. These are close to atoms with large apparent thermal motions.

```
PLAT242_ALERT_2_C Low    'MainMol' Ueq as Compared to Neighbors of        S23 Check
```

Author Response: The Ag07, Ag19, S23, S25, S27, S36, S37, S38, S40, S42, P2, P3, P4, P6, P8, P10, P20, C12I and C15J show no disorder and cannot be elements different to those attributed. These are close to atoms with large apparent thermal motions.

```
PLAT242_ALERT_2_C Low    'MainMol' Ueq as Compared to Neighbors of        S25 Check
```

Author Response: The Ag07, Ag19, S23, S25, S27, S36, S37, S38, S40, S42, P2, P3, P4, P6, P8, P10, P20, C12I and C15J show no disorder and cannot be elements different to those attributed. These are close to atoms with large apparent thermal motions.

```
PLAT242_ALERT_2_C Low    'MainMol' Ueq as Compared to Neighbors of        S27 Check
```

Author Response: The Ag07, Ag19, S23, S25, S27, S36, S37, S38, S40, S42, P2, P3, P4, P6, P8, P10, P20, C12I and C15J show no disorder and cannot be elements different to those attributed. These are close to atoms with large apparent thermal motions.

```
PLAT242_ALERT_2_C Low    'MainMol' Ueq as Compared to Neighbors of        S36 Check
```

Author Response: The Ag07, Ag19, S23, S25, S27, S36, S37, S38, S40, S42, P2, P3, P4, P6, P8, P10, P20, C12I and C15J show no disorder and cannot be elements different to those attributed. These are close to atoms with large apparent thermal motions.

```
PLAT242_ALERT_2_C Low    'MainMol' Ueq as Compared to Neighbors of        S37 Check
```

Author Response: The Ag07, Ag19, S23, S25, S27, S36, S37, S38, S40, S42, P2, P3, P4, P6, P8, P10, P20, C12I and C15J show no disorder and cannot be elements different to those attributed. These are close to atoms with large apparent thermal motions.

PLAT242_ALERT_2_C Low 'MainMol' Ueq as Compared to Neighbors of S38 Check

Author Response: The Ag07, Ag19, S23, S25, S27, S36, S37, S38, S40, S42, P2, P3, P4, P6, P8, P10, P20, C12I and C15J show no disorder and cannot be elements different to those attributed. These are close to atoms with large apparent thermal motions.

PLAT242_ALERT_2_C Low 'MainMol' Ueq as Compared to Neighbors of S40 Check

Author Response: The Ag07, Ag19, S23, S25, S27, S36, S37, S38, S40, S42, P2, P3, P4, P6, P8, P10, P20, C12I and C15J show no disorder and cannot be elements different to those attributed. These are close to atoms with large apparent thermal motions.

PLAT242_ALERT_2_C Low 'MainMol' Ueq as Compared to Neighbors of S42 Check

Author Response: The Ag07, Ag19, S23, S25, S27, S36, S37, S38, S40, S42, P2, P3, P4, P6, P8, P10, P20, C12I and C15J show no disorder and cannot be elements different to those attributed. These are close to atoms with large apparent thermal motions.

PLAT242_ALERT_2_C Low 'MainMol' Ueq as Compared to Neighbors of P2 Check

Author Response: The Ag07, Ag19, S23, S25, S27, S36, S37, S38, S40, S42, P2, P3, P4, P6, P8, P10, P20, C12I and C15J show no disorder and cannot be elements different to those attributed. These are close to atoms with large apparent thermal motions.

PLAT242_ALERT_2_C Low 'MainMol' Ueq as Compared to Neighbors of P3 Check

Author Response: The Ag07, Ag19, S23, S25, S27, S36, S37, S38, S40, S42, P2, P3, P4, P6, P8, P10, P20, C12I and C15J show no disorder and cannot be elements different to those attributed. These are close to atoms with large apparent thermal motions.

PLAT242_ALERT_2_C Low 'MainMol' Ueq as Compared to Neighbors of P4 Check

Author Response: The Ag07, Ag19, S23, S25, S27, S36, S37, S38, S40, S42, P2, P3, P4, P6, P8, P10, P20, C12I and C15J show no disorder and cannot be elements different to those attributed. These are close to atoms with large apparent thermal motions.

PLAT242_ALERT_2_C Low 'MainMol' Ueq as Compared to Neighbors of P6 Check

Author Response: The Ag07, Ag19, S23, S25, S27, S36, S37, S38, S40, S42, P2, P3, P4, P6, P8, P10, P20, C12I and C15J show no disorder and cannot be elements different to those attributed. These are close to atoms with large apparent thermal motions.

PLAT242_ALERT_2_C Low 'MainMol' Ueq as Compared to Neighbors of P8 Check

Author Response: The Ag07, Ag19, S23, S25, S27, S36, S37, S38, S40, S42, P2, P3, P4, P6, P8, P10, P20, C12I and C15J show no disorder and cannot be elements different to those attributed. These are close to atoms with large apparent thermal motions.

PLAT242_ALERT_2_C Low 'MainMol' Ueq as Compared to Neighbors of P10 Check

Author Response: The Ag07, Ag19, S23, S25, S27, S36, S37, S38, S40, S42, P2, P3, P4, P6, P8, P10, P20, C12I and C15J show no disorder and cannot be elements different to those attributed. These are close to atoms with large apparent thermal motions.

PLAT242_ALERT_2_C Low 'MainMol' Ueq as Compared to Neighbors of P20 Check

Author Response: The Ag07, Ag19, S23, S25, S27, S36, S37, S38, S40, S42, P2, P3, P4, P6, P8, P10, P20, C12I and C15J show no disorder and cannot be elements different to those attributed. These are close to atoms with large apparent thermal motions.

PLAT242_ALERT_2_C Low 'MainMol' Ueq as Compared to Neighbors of C12I Check

Author Response: The Ag07, Ag19, S23, S25, S27, S36, S37, S38, S40, S42, P2, P3, P4, P6, P8, P10, P20, C12I and C15J show no disorder and cannot be elements different to those attributed. These are close to atoms with large apparent thermal motions.

PLAT242_ALERT_2_C Low 'MainMol' Ueq as Compared to Neighbors of C15J Check

Author Response: The Ag07, Ag19, S23, S25, S27, S36, S37, S38, S40, S42, P2, P3, P4, P6, P8, P10, P20, C12I and C15J show no disorder and cannot be elements different to those attributed. These are close to atoms with large apparent thermal motions.

```
PLAT410_ALERT_2_C Short  Intra H...H Contact  H4F       ..H4L       .       1.96 Ang.
                                                       x,y,z  =      1_555 Check
```

Author Response: The close contact is between two H atoms of symmetry related phenyl groups. These is no doubt of their positions.

```
PLAT721_ALERT_1_C Bond    Calc     1.41(7), Rep     1.39000 Dev...       0.02 Ang.
              C10I     -C10J           1_555   1_555 ........     #  585 Check
```

Author Response: All phenyl groups were refined using AFIX 66 with the standard C-C distance of 1.39 \%A.

```
PLAT906_ALERT_3_C Large K Value in the Analysis of Variance ......     22.399 Check
```

Author Response: The large value of K (average Fo/Fc), 21.045, occurs for the Fc/Fc(max) bin between 0.000-0.009, which are the weakest reflections is is a result of the very high noise floor of the Photon 100 detector and the possibility of unmodelled electron density, though SQUEEZE was used to remove the diffuse eletron density of the cations and solvent molecules.

```
PLAT906_ALERT_3_C Large K Value in the Analysis of Variance ......      5.755 Check
```

Author Response: The large value of K (average Fo/Fc), 21.045, occurs for the Fc/Fc(max) bin between 0.000-0.009, which are the weakest reflections is is a result of the very high noise floor of the Photon 100 detector and the possibility of unmodelled electron density, though SQUEEZE was used to remove the diffuse eletron density of the cations and solvent molecules.

```
PLAT906_ALERT_3_C Large K Value in the Analysis of Variance ......      3.093 Check
```

Author Response: The large value of K (average Fo/Fc), 21.045, occurs for the Fc/Fc(max) bin between 0.000-0.009, which are the weakest reflections is is a result of the very high noise floor of the Photon 100 detector and the possibility of unmodelled electron density, though SQUEEZE was used to remove the diffuse eletron density of the cations and solvent molecules.

```
PLAT906_ALERT_3_C Large K Value in the Analysis of Variance ......      2.178 Check
```

Author Response: The large value of K (average Fo/Fc), 21.045, occurs for the Fc/Fc(max) bin between 0.000-0.009, which are the weakest reflections is is a result of the very high noise floor of the Photon 100 detector and the possibility of unmodelled electron density, though SQUEEZE was used to remove the diffuse eletron density of the cations and solvent molecules.

```
PLAT911_ALERT_3_C Missing FCF Refl Between Thmin & STh/L=    0.600         12 Report
             -17  3  3,   1  0  4, -11  1  5,   1  7  6,   6  6  6,   5  7  7,
              -7 13  9, -13  1 10,  -4  2 12,   3  0 12,   6  9 14,  -9  2 15,
```

Author Response: These reflections were measured but were eliminated by SAINT for exceeding the image queue.

```
PLAT913_ALERT_3_C Missing # of Very Strong Reflections in FCF ....          5 Note
                2  0  0,  -1  1  1,   1  1  1,  -1  0  2,   0  0  2,
```

Author Response: These reflections were occluded by the beam stop.

```
PLAT918_ALERT_3_C Reflection(s) with I(obs) much Smaller I(calc) .         10 Check
```

Author Response: These reflection might have been partially occluded by the beam stop or were measured at the edge of the detector ou under the central wires of one of the CMOS panels.

```
PLAT977_ALERT_2_C Check Negative Difference Density on H1C       .      -0.60 eA-3
```

Author Response: Many H atoms had negative difference electron density. It might be due to the PLATON SQUEEZE routine.

```
PLAT977_ALERT_2_C Check Negative Difference Density on H1D       .      -0.70 eA-3
```

Author Response: Many H atoms had negative difference electron density. It might be due to the PLATON SQUEEZE routine.

```
PLAT977_ALERT_2_C Check Negative Difference Density on H1I       .      -0.92 eA-3
```

Author Response: Many H atoms had negative difference electron density. It might be due to the PLATON SQUEEZE routine.

```
PLAT977_ALERT_2_C Check Negative Difference Density on H1J       .      -0.49 eA-3
```

Author Response: Many H atoms had negative difference electron density. It might be due to the PLATON SQUEEZE routine.

```
PLAT977_ALERT_2_C Check Negative Difference Density on H1K       .      -0.81 eA-3
```

Author Response: Many H atoms had negative difference electron density. It might be due to the PLATON SQUEEZE routine.

PLAT977_ALERT_2_C Check Negative Difference Density on H1L . -0.70 eA-3

Author Response: Many H atoms had negative difference electron density. It might be due to the PLATON SQUEEZE routine.

PLAT977_ALERT_2_C Check Negative Difference Density on H2C . -0.46 eA-3

Author Response: Many H atoms had negative difference electron density. It might be due to the PLATON SQUEEZE routine.

PLAT977_ALERT_2_C Check Negative Difference Density on H2D . -0.65 eA-3

Author Response: Many H atoms had negative difference electron density. It might be due to the PLATON SQUEEZE routine.

PLAT977_ALERT_2_C Check Negative Difference Density on H2E . -0.40 eA-3

Author Response: Many H atoms had negative difference electron density. It might be due to the PLATON SQUEEZE routine.

PLAT977_ALERT_2_C Check Negative Difference Density on H2F . -0.85 eA-3

Author Response: Many H atoms had negative difference electron density. It might be due to the PLATON SQUEEZE routine.

PLAT977_ALERT_2_C Check Negative Difference Density on H2J . -1.22 eA-3

Author Response: Many H atoms had negative difference electron density. It might be due to the PLATON SQUEEZE routine.

PLAT977_ALERT_2_C Check Negative Difference Density on H3I . -0.57 eA-3

Author Response: Many H atoms had negative difference electron density. It might be due to the PLATON SQUEEZE routine.

PLAT977_ALERT_2_C Check Negative Difference Density on H3J . -0.99 eA-3

Author Response: Many H atoms had negative difference electron density. It might be due to the PLATON SQUEEZE routine.

PLAT977_ALERT_2_C Check Negative Difference Density on H3L . -0.47 eA-3

Author Response: Many H atoms had negative difference electron density. It might be due to the PLATON SQUEEZE routine.

```
PLAT977_ALERT_2_C Check Negative Difference Density on H4F       .      -0.70 eA-3
```

Author Response: Many H atoms had negative difference electron density. It might be due to the PLATON SQUEEZE routine.

```
PLAT977_ALERT_2_C Check Negative Difference Density on H4L       .      -0.81 eA-3
```

Author Response: Many H atoms had negative difference electron density. It might be due to the PLATON SQUEEZE routine.

```
PLAT977_ALERT_2_C Check Negative Difference Density on H5B       .      -0.70 eA-3
```

Author Response: Many H atoms had negative difference electron density. It might be due to the PLATON SQUEEZE routine.

```
PLAT977_ALERT_2_C Check Negative Difference Density on H5C       .      -0.37 eA-3
```

Author Response: Many H atoms had negative difference electron density. It might be due to the PLATON SQUEEZE routine.

```
PLAT977_ALERT_2_C Check Negative Difference Density on H5E       .      -0.72 eA-3
```

Author Response: Many H atoms had negative difference electron density. It might be due to the PLATON SQUEEZE routine.

```
PLAT977_ALERT_2_C Check Negative Difference Density on H5I       .      -0.44 eA-3
```

Author Response: Many H atoms had negative difference electron density. It might be due to the PLATON SQUEEZE routine.

```
PLAT977_ALERT_2_C Check Negative Difference Density on H6D       .      -0.59 eA-3
```

Author Response: Many H atoms had negative difference electron density. It might be due to the PLATON SQUEEZE routine.

```
PLAT977_ALERT_2_C Check Negative Difference Density on H6K       .      -0.49 eA-3
```

Author Response: Many H atoms had negative difference electron density. It might be due to the PLATON SQUEEZE routine.

```
PLAT977_ALERT_2_C Check Negative Difference Density on H7B       .      -1.45 eA-3
```

Author Response: Many H atoms had negative difference electron density. It might be due to the PLATON SQUEEZE routine.

```
PLAT977_ALERT_2_C Check Negative Difference Density on H7F       .      -0.73 eA-3
```

Author Response: Many H atoms had negative difference electron density. It might be due to the PLATON SQUEEZE routine.

```
PLAT977_ALERT_2_C Check Negative Difference Density on H7H       .      -0.67 eA-3
```

Author Response: Many H atoms had negative difference electron density. It might be due to the PLATON SQUEEZE routine.

```
PLAT977_ALERT_2_C Check Negative Difference Density on H7K       .      -1.01 eA-3
```

Author Response: Many H atoms had negative difference electron density. It might be due to the PLATON SQUEEZE routine.

```
PLAT977_ALERT_2_C Check Negative Difference Density on H7L       .      -0.40 eA-3
```

Author Response: Many H atoms had negative difference electron density. It might be due to the PLATON SQUEEZE routine.

```
PLAT977_ALERT_2_C Check Negative Difference Density on H8E       .      -0.86 eA-3
```

Author Response: Many H atoms had negative difference electron density. It might be due to the PLATON SQUEEZE routine.

```
PLAT977_ALERT_2_C Check Negative Difference Density on H8J       .      -1.07 eA-3
```

Author Response: Many H atoms had negative difference electron density. It might be due to the PLATON SQUEEZE routine.

```
PLAT977_ALERT_2_C Check Negative Difference Density on H9B       .      -0.33 eA-3
```

Author Response: Many H atoms had negative difference electron density. It might be due to the PLATON SQUEEZE routine.

```
PLAT977_ALERT_2_C Check Negative Difference Density on H9E       .      -0.41 eA-3
```

Author Response: Many H atoms had negative difference electron density. It might be due to the PLATON SQUEEZE routine.

PLAT977_ALERT_2_C Check Negative Difference Density on H10C . -0.35 eA-3

Author Response: Many H atoms had negative difference electron density. It might be due to the PLATON SQUEEZE routine.

PLAT977_ALERT_2_C Check Negative Difference Density on H10E . -0.61 eA-3

Author Response: Many H atoms had negative difference electron density. It might be due to the PLATON SQUEEZE routine.

PLAT977_ALERT_2_C Check Negative Difference Density on H10F . -0.47 eA-3

Author Response: Many H atoms had negative difference electron density. It might be due to the PLATON SQUEEZE routine.

PLAT977_ALERT_2_C Check Negative Difference Density on H10H . -0.57 eA-3

Author Response: Many H atoms had negative difference electron density. It might be due to the PLATON SQUEEZE routine.

PLAT977_ALERT_2_C Check Negative Difference Density on H11F . -0.45 eA-3

Author Response: Many H atoms had negative difference electron density. It might be due to the PLATON SQUEEZE routine.

PLAT977_ALERT_2_C Check Negative Difference Density on H11I . -0.35 eA-3

Author Response: Many H atoms had negative difference electron density. It might be due to the PLATON SQUEEZE routine.

PLAT977_ALERT_2_C Check Negative Difference Density on H12C . -0.65 eA-3

Author Response: Many H atoms had negative difference electron density. It might be due to the PLATON SQUEEZE routine.

PLAT977_ALERT_2_C Check Negative Difference Density on H12D . -0.47 eA-3

Author Response: Many H atoms had negative difference electron density. It might be due to the PLATON SQUEEZE routine.

PLAT977_ALERT_2_C Check Negative Difference Density on H12F . -1.11 eA-3

Author Response: Many H atoms had negative difference electron density. It might be due to the PLATON SQUEEZE routine.

PLAT977_ALERT_2_C Check Negative Difference Density on H12H . -0.62 eA-3

Author Response: Many H atoms had negative difference electron density. It might be due to the PLATON SQUEEZE routine.

PLAT977_ALERT_2_C Check Negative Difference Density on H12L . -1.22 eA-3

Author Response: Many H atoms had negative difference electron density. It might be due to the PLATON SQUEEZE routine.

PLAT977_ALERT_2_C Check Negative Difference Density on H13C . -0.92 eA-3

Author Response: Many H atoms had negative difference electron density. It might be due to the PLATON SQUEEZE routine.

PLAT977_ALERT_2_C Check Negative Difference Density on H13H . -0.48 eA-3

Author Response: Many H atoms had negative difference electron density. It might be due to the PLATON SQUEEZE routine.

PLAT977_ALERT_2_C Check Negative Difference Density on H14E . -0.57 eA-3

Author Response: Many H atoms had negative difference electron density. It might be due to the PLATON SQUEEZE routine.

PLAT977_ALERT_2_C Check Negative Difference Density on H14H . -0.54 eA-3

Author Response: Many H atoms had negative difference electron density. It might be due to the PLATON SQUEEZE routine.

PLAT977_ALERT_2_C Check Negative Difference Density on H15E . -0.39 eA-3

Author Response: Many H atoms had negative difference electron density. It might be due to the PLATON SQUEEZE routine.

PLAT977_ALERT_2_C Check Negative Difference Density on H15F . -0.55 eA-3

Author Response: Many H atoms had negative difference electron density. It might be due to the PLATON SQUEEZE routine.

```
PLAT977_ALERT_2_C Check Negative Difference Density on H15H      .      -0.42 eA-3
```

Author Response: Many H atoms had negative difference electron density. It might be due to the PLATON SQUEEZE routine.

```
PLAT977_ALERT_2_C Check Negative Difference Density on H15I      .      -0.68 eA-3
```

Author Response: Many H atoms had negative difference electron density. It might be due to the PLATON SQUEEZE routine.

```
PLAT977_ALERT_2_C Check Negative Difference Density on H15K      .      -1.15 eA-3
```

Author Response: Many H atoms had negative difference electron density. It might be due to the PLATON SQUEEZE routine.

```
PLAT977_ALERT_2_C Check Negative Difference Density on H16C      .      -0.65 eA-3
```

Author Response: Many H atoms had negative difference electron density. It might be due to the PLATON SQUEEZE routine.

```
PLAT977_ALERT_2_C Check Negative Difference Density on H16H      .      -0.71 eA-3
```

Author Response: Many H atoms had negative difference electron density. It might be due to the PLATON SQUEEZE routine.

```
PLAT977_ALERT_2_C Check Negative Difference Density on H16I      .      -0.51 eA-3
```

Author Response: Many H atoms had negative difference electron density. It might be due to the PLATON SQUEEZE routine.

```
PLAT977_ALERT_2_C Check Negative Difference Density on H17F      .      -0.54 eA-3
```

Author Response: Many H atoms had negative difference electron density. It might be due to the PLATON SQUEEZE routine.

```
PLAT977_ALERT_2_C Check Negative Difference Density on H17I      .      -0.50 eA-3
```

Author Response: Many H atoms had negative difference electron density. It might be due to the PLATON SQUEEZE routine.

PLAT977_ALERT_2_C Check Negative Difference Density on H19F . -0.64 eA-3

Author Response: Many H atoms had negative difference electron density. It might be due to the PLATON SQUEEZE routine.

PLAT977_ALERT_2_C Check Negative Difference Density on H19K . -0.40 eA-3

Author Response: Many H atoms had negative difference electron density. It might be due to the PLATON SQUEEZE routine.

PLAT977_ALERT_2_C Check Negative Difference Density on H19L . -0.78 eA-3

Author Response: Many H atoms had negative difference electron density. It might be due to the PLATON SQUEEZE routine.

PLAT977_ALERT_2_C Check Negative Difference Density on H20E . -0.61 eA-3

Author Response: Many H atoms had negative difference electron density. It might be due to the PLATON SQUEEZE routine.

PLAT977_ALERT_2_C Check Negative Difference Density on H20F . -0.34 eA-3

Author Response: Many H atoms had negative difference electron density. It might be due to the PLATON SQUEEZE routine.

PLAT977_ALERT_2_C Check Negative Difference Density on H20I . -0.90 eA-3

Author Response: Many H atoms had negative difference electron density. It might be due to the PLATON SQUEEZE routine.

PLAT977_ALERT_2_C Check Negative Difference Density on H21C . -1.49 eA-3

Author Response: Many H atoms had negative difference electron density. It might be due to the PLATON SQUEEZE routine.

PLAT977_ALERT_2_C Check Negative Difference Density on H21D . -0.55 eA-3

Author Response: Many H atoms had negative difference electron density. It might be due to the PLATON SQUEEZE routine.

PLAT977_ALERT_2_C Check Negative Difference Density on H21I . -0.77 eA-3

Author Response: Many H atoms had negative difference electron density. It might be due to the PLATON SQUEEZE routine.

```
PLAT977_ALERT_2_C Check Negative Difference Density on H22B      .      -0.69 eA-3
```

Author Response: Many H atoms had negative difference electron density. It might be due to the PLATON SQUEEZE routine.

```
PLAT977_ALERT_2_C Check Negative Difference Density on H22H      .      -0.76 eA-3
```

Author Response: Many H atoms had negative difference electron density. It might be due to the PLATON SQUEEZE routine.

```
PLAT977_ALERT_2_C Check Negative Difference Density on H22J      .      -0.62 eA-3
```

Author Response: Many H atoms had negative difference electron density. It might be due to the PLATON SQUEEZE routine.

```
PLAT977_ALERT_2_C Check Negative Difference Density on H22K      .      -0.31 eA-3
```

Author Response: Many H atoms had negative difference electron density. It might be due to the PLATON SQUEEZE routine.

```
PLAT977_ALERT_2_C Check Negative Difference Density on H23C      .      -0.63 eA-3
```

Author Response: Many H atoms had negative difference electron density. It might be due to the PLATON SQUEEZE routine.

```
PLAT977_ALERT_2_C Check Negative Difference Density on H23F      .      -0.39 eA-3
```

Author Response: Many H atoms had negative difference electron density. It might be due to the PLATON SQUEEZE routine.

```
PLAT977_ALERT_2_C Check Negative Difference Density on H23I      .      -0.98 eA-3
```

Author Response: Many H atoms had negative difference electron density. It might be due to the PLATON SQUEEZE routine.

```
PLAT977_ALERT_2_C Check Negative Difference Density on H24B      .      -1.22 eA-3
```

Author Response: Many H atoms had negative difference electron density. It might be due to the PLATON SQUEEZE routine.

```
PLAT977_ALERT_2_C Check Negative Difference Density on H24D      .      -0.83 eA-3
```

Author Response: Many H atoms had negative difference electron density. It might be due to the PLATON SQUEEZE routine.

Alert level G

```
PLAT003_ALERT_2_G Number of Uiso or U(i,j)  Restrained non-H-Atoms       312 Report
PLAT083_ALERT_2_G SHELXL Second Parameter in WGHT  Unusually Large    7252.48 Why ?
PLAT178_ALERT_4_G The CIF-Embedded .res File Contains SIMU Records         2 Report
PLAT180_ALERT_4_G Check Cell Rounding: # of Values Ending with 0 =         3 Note
PLAT186_ALERT_4_G The CIF-Embedded .res File Contains ISOR Records         1 Report
PLAT187_ALERT_4_G The CIF-Embedded .res File Contains RIGU Records         1 Report
PLAT188_ALERT_3_G A Non-default SIMU Restraint Value has been used     0.0200 Report
PLAT301_ALERT_3_G Main Residue  Disorder .............(Resd    1)         4% Note
PLAT432_ALERT_2_G Short Inter X...Y Contact  C4E      ..C4E      .      2.87 Ang.
                                                1-x,y,1/2-z  =      2_655 Check
PLAT432_ALERT_2_G Short Inter X...Y Contact  C20C     ..C20C     .      3.17 Ang.
                                                2-x,1-y,1-z  =      3_766 Check
PLAT606_ALERT_4_G Solvent Accessible VOID(S) in  Crystal Structure         ! Info
PLAT720_ALERT_4_G Number of Unusual/Non-Standard Labels ..........         9 Note
              Ag01    Ag02    Ag03    Ag04    Ag05    Ag06    Ag07    Ag08
              Ag09
PLAT779_ALERT_4_G Suspect or Irrelevant (Bond) Angle(s) in CIF ...      33.80 Deg.
              S44  -S25  -AG51    1_555   1_555   1_555 ......     # 1661 Check
PLAT779_ALERT_4_G Suspect or Irrelevant (Bond) Angle(s) in CIF ...      40.80 Deg.
              AG63 -S28  -AG21    1_555   1_555   1_555 ......     # 1747 Check
PLAT779_ALERT_4_G Suspect or Irrelevant (Bond) Angle(s) in CIF ...      35.40 Deg.
              S18  -P18  -AG60    1_555   1_555   1_555 ......     # 2760 Check
PLAT860_ALERT_3_G Number of Least-Squares Restraints ............       5310 Note
PLAT869_ALERT_4_G ALERTS Related to the Use of SQUEEZE  Suppressed         ! Info
PLAT912_ALERT_4_G Missing # of FCF Reflections Above STh/L=  0.600       466 Note
PLAT969_ALERT_5_G The 'Henn et al.' R-Factor-gap value ..........      6.938 Note
              Predicted wR2: Based on SigI**2  6.74 or SHELX Weight 43.05
PLAT978_ALERT_2_G Number C-C Bonds with Positive Residual Density.         0 Info
```

```
  34 ALERT level A = Most likely a serious problem - resolve or explain
  33 ALERT level B = A potentially serious problem, consider carefully
 141 ALERT level C = Check. Ensure it is not caused by an omission or oversight
  20 ALERT level G = General information/check it is not something unexpected

   4 ALERT type 1 CIF construction/syntax error, inconsistent or missing data
 195 ALERT type 2 Indicator that the structure model may be wrong or deficient
  17 ALERT type 3 Indicator that the structure quality may be low
  11 ALERT type 4 Improvement, methodology, query or suggestion
   1 ALERT type 5 Informative message, check
```

---

checkCIF publication errors

Alert level A

```
PUBL006_ALERT_1_A  _publ_requested_journal is missing
            e.g. 'Acta Crystallographica Section C'
```

```
   1 ALERT level A = Data missing that is essential or data in wrong format
   0 ALERT level G = General alerts. Data that may be required is missing
```

Publication of your CIF

You should attempt to resolve as many as possible of the alerts in all categories. Often the minor alerts point to easily fixed oversights, errors and omissions in your CIF or refinement strategy, so attention to these fine details can be worthwhile. In order to resolve some of the more serious problems it may be necessary to carry out additional measurements or structure refinements. However, the nature of your study may justify the reported deviations from journal submission requirements and the more serious of these should be commented upon in the discussion or experimental section of a paper or in the "special_details" fields of the CIF. *checkCIF* was carefully designed to identify outliers and unusual parameters, but every test has its limitations and alerts that are not important in a particular case may appear. Conversely, the absence of alerts does not guarantee there are no aspects of the results needing attention. It is up to the individual to critically assess their own results and, if necessary, seek expert advice.

If level A alerts remain, which you believe to be justified deviations, and you intend to submit this CIF for publication in a journal, you should additionally insert an explanation in your CIF using the Validation Reply Form (VRF) below. This will allow your explanation to be considered as part of the review process.

Validation response form

Please find below a validation response form (VRF) that can be filled in and pasted into your CIF.

```
# start Validation Reply Form
_vrf_PUBL006_GLOBAL
;
PROBLEM: _publ_requested_journal is missing
RESPONSE: ...
;
_vrf_PLAT971_RB_UFRGS_HS_GM79_100K_Mo
;
PROBLEM: Check Calcd Resid. Dens.  1.24Ang From Ag21            8.58 eA-3
RESPONSE: ...
;
_vrf_PLAT972_RB_UFRGS_HS_GM79_100K_Mo
;
PROBLEM: Check Calcd Resid. Dens.  1.10Ang From Ag38           -5.05 eA-3
RESPONSE: ...
;
_vrf_PLAT048_RB_UFRGS_HS_GM79_100K_Mo
;
```

```
PROBLEM: MoietyFormula Not Given (or Incomplete) ........     Please Check
RESPONSE: ...
;
# end Validation Reply Form
```

If you wish to submit your CIF for publication in Acta Crystallographica Section C or E, you should upload your CIF via the web. If you wish to submit your CIF for publication in IUCrData you should upload your CIF via the web. If your CIF is to form part of a submission to another IUCr journal, you will be asked, either during electronic submission or by the Co-editor handling your paper, to upload your CIF via our web site.

PLATON version of 04/06/2025; check.def file version of 30/05/2025

---

duplicate check

No duplication found

Datablock RB_UFRGS_HS_GM79_100K_Mo - ellipsoid plot

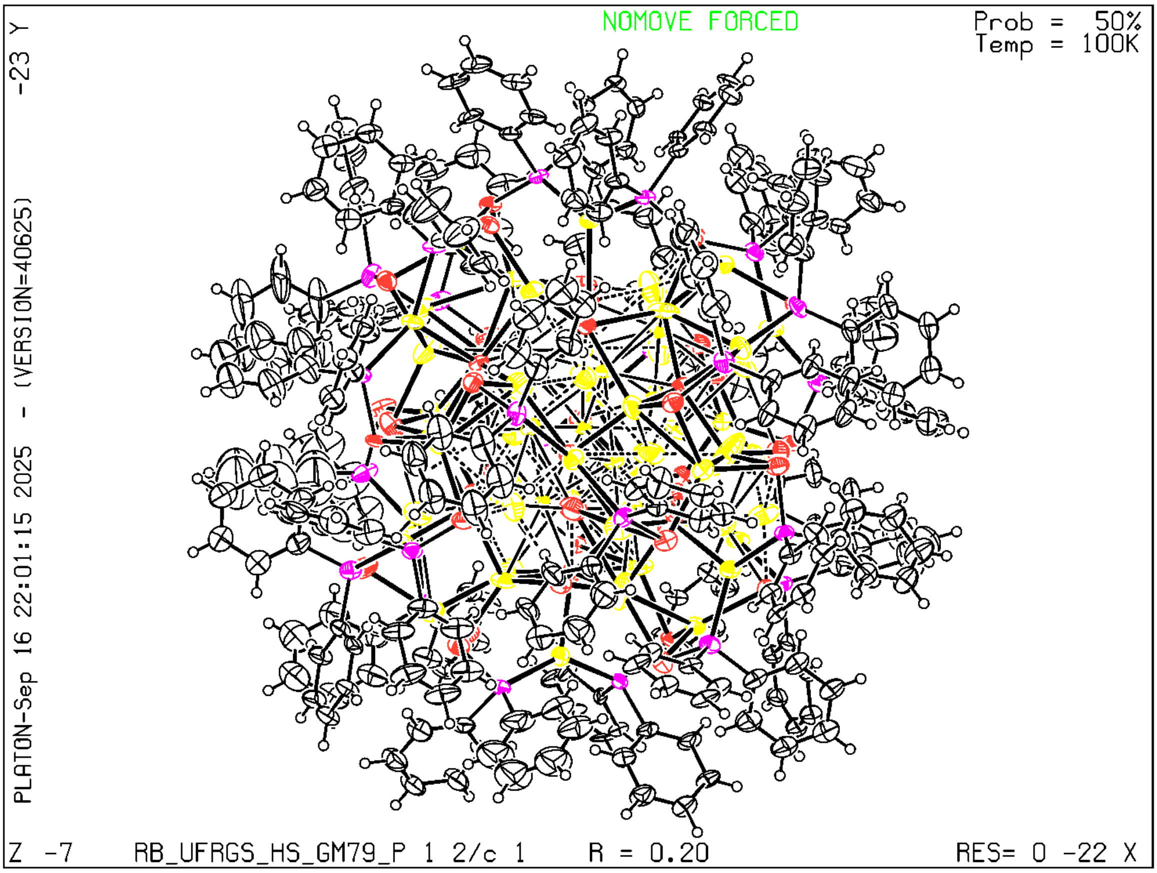

Supplement: Supplementary file 8 [file ao5c08304_si_008.pdf]
